# Yam Gruel alone and in combination with metformin regulates hepatic lipid metabolism disorders in a diabetic rat model by activating the AMPK/ACC/CPT-1 pathway

**DOI:** 10.1186/s12944-024-02014-2

**Published:** 2024-01-25

**Authors:** Yanling Dai, Chen Qiu, Diandian Zhang, Mianli Li, Weinan Liu

**Affiliations:** 1 School of Nursing, Fujian University of Traditional Chinese Medicine, Fuzhou, 350122 China; 2 Department of Endocrine, People’s Hospital Affiliated to Fujian University of Traditional Chinese Medicine, Fuzhou, 350004 China; 3 Department of Orthopedics, People’s Hospital Affiliated to Fujian University of Traditional Chinese Medicine, Fuzhou, 350004 China

**Keywords:** Yam Gruel, Diet therapy, Type 2 diabetes, Dyslipidaemia, Liver, AMPK signalling pathway

## Abstract

**Background:**

As independent and correctable risk factors, disturbances in lipid metabolism are significantly associated with type 2 diabetes mellitus (T2DM). This research investigated the mechanism underlying the lipid-regulating effects of Yam Gruel in diabetic rats.

**Methods:**

First, rats in the control group were given a normal diet, and a diabetic rat model was established via the consumption of a diet that was rich in both fat and sugar for six weeks followed by the intraperitoneal injection of streptozotocin (STZ). After the model was established, the rats were divided into five distinct groups: the control group, model group, Yam Gruel (SYZ) group, metformin (MET) group, and combined group; each treatment was administered for six weeks. The fasting blood glucose (FBG), body and liver weights as well as liver index of the rats were determined. Total cholesterol (TC), triglyceride (TG), high-density lipoprotein cholesterol (HDL-C), low-density lipoprotein cholesterol (LDL-C), aspartic acid transaminase (AST), alanine aminotransferase (ALT), and nonesterified fatty acid (NEFA) levels were measured. Oil Red O staining was used to assess hepatic steatosis. In addition, the levels of Phospho-acetyl-CoA carboxylase (p-ACC), acetyl coenzyme A carboxylase (ACC), AMP-activated protein kinase (AMPK), Phospho-AMPK (p-AMPK), carnitine palmitoyl transferase I (CPT-1), and Malonyl-CoA decarboxylase (MLYCD) in liver tissues were measured by real-time PCR (q-PCR) and western blotting.

**Results:**

After 6 weeks of treatment, Yam Gruel alone or in combination with metformin significantly reduced FBG level, liver weight and index. The concentrations of lipid indices (TG, TC, NEFA, and LDL-C), the levels of liver function indices (ALT and AST) and the degree of hepatic steatosis was improved in diabetic rats that were treated with Yam Gruel with or without metformin. Furthermore, Yam Gruel increased the protein levels of p-ACC/ACC, p-AMPK/AMPK, MLYCD, and CPT-1, which was consistent with the observed changes in gene expression. Additionally, the combination of these two agents was significantly more effective in upregulating the expression of AMPK pathway-related genes and proteins.

**Conclusions:**

These results demonstrated that Yam Gruel may be a potential diet therapy for improving lipid metabolism in T2DM patients and that it may exert its effects via AMPK/ACC/CPT-1 pathway activation. In some respects, the combination of Yam Gruel and metformin exerted more benefits effects than Yam Gruel alone.

**Supplementary Information:**

The online version contains supplementary material available at 10.1186/s12944-024-02014-2.

## Introduction

Diabetes is among the most rapidly growing health problems of the 21^st^ century, as shown by a substantial increase in the prevalence of this condition, which tripled over the previous two decades. The International Diabetes Federation (IDF) recently published the 10th edition of the Diabetes Atlas, which revealed that 537 million individuals, or 10.5% of the population aged between 20 and 79 years, live with diabetes [1]. An exponential increase in the numbers of patients with T2DM in developing countries, including China, has been observed owing to the ageing of the population and cultural changes [2]. Based on the Chinese guidelines for the prevention and management of T2DM (2020 Edition) [3], the prevalence of this disease in China was estimated to be 11.2%, indicating a sustained increasing trend. Persistent increases in blood sugar levels can lead to significant disorders that affect the liver, heart, blood vessels, kidneys, and nerves. The development of diabetes and its complications increases the need for medical care, diminishes quality of life (QoL), and results in excessive burdens on families [4, 5]. Consequently, T2DM imposes a substantial burden on health care resources, and effective therapies need to be developed to combat the T2DM epidemic.

Dyslipidaemia is a distinct and reversible risk factor for T2DM, and individuals suffering from T2DM exhibit impairments in lipid metabolism. There is a correlation between dyslipidaemia and hyperglycaemia. In diabetic patients, inadequate insulin levels contribute to a decrease in the activity of enzymes that are associated with lipid metabolism, leading to dyslipidaemia. Simultaneously, dyslipidaemia induces insulin resistance, impaired insulin secretion, and β-cell apoptosis [6, 7]. Previous studies demonstrated that more than half of T2DM patients exhibit dyslipidaemia, which is a condition that involves increased concentrations of triglyceride (TG), total cholesterol (TC), and low-density lipoprotein cholesterol (LDL-C) or decreased concentrations of high-density lipoprotein cholesterol (HDL-C) [8]. The liver plays a pivotal role in lipid metabolism, serving as the central site for fatty acid synthesis and the circulation of lipids through lipoprotein synthesis. Hepatic lipid metabolism is mediated by several essential genes and nuclear factors, including fatty acid-binding proteins (FABPs), CD36, and TM6SF2, which regulate processes such as free fatty acid oxidation, lipid oxidation, and the synthesis of very low-density lipoproteins in hepatic cells [9, 10]. A multicentre nationwide survey of 4807 Chinese adult patients from 20 endocrinology clinics in China revealed a considerable incidence of dyslipidaemia (67.1%) [11]. Therefore, the effect of dyslipidaemia on the development and progression of T2DM should not be overlooked, and effective methods should be developed to ameliorate dyslipidaemia in T2DM patients. Currently, statins are widely considered to be primary lipid-lowering medications, but the use of statins is influenced by many factors, including safety, tolerability, and patient adherence [12, 13]. Chinese individuals have poor tolerance for high-intensity statin therapy, and are more susceptible to hepatotoxicity and muscle symptoms than individuals in Europe and North America, even after the long-term administration of statins, which leads to fluctuations in blood glucose levels [14]. Over the previous ten years, a number of lipid-lowering medications, such as anti-PCSK9 monoclonal antibodies, that are not statins have received increasing attention, but most of these new therapies are currently being tested in experimental or clinical trials; additionally, they are not widely used due to their high cost and inconvenient delivery methods [15, 16]. Therefore, the current guidelines and expert consensus clearly highlight that promoting healthy adjustments to diet and lifestyle is the primary therapeutic approach for managing dyslipidaemia in patients with T2DM [14, 17–19].

Nutritional therapy is the main approach that is used to treat patients with T2DM, and it plays a pivotal role in the comprehensive regulation of diabetes, even when medication has been initiated. The inclusion of nutritional therapy in the management of diabetes has demonstrated favourable outcomes, including weight reduction, favourable glycaemic control, and decreases in blood pressure, lipid levels, and glycosylated haemoglobin levels [20–24]. Based on the 2019 publication titled “Nutrition Therapy for Adults with Diabetes or Prediabetes: A Consensus Report,” various dietary regimens, such as low-carbohydrate diets, Mediterranean diets, and low-fat vegan diets, are appropriate for T2DM management [25–28].

However, it is important to note that there is no ideal “one-size-fits-all” diet for diabetic patients worldwide, and planning meals must be tailored according to subjective and cultural preferences, resources, and health goals. Rice is an important carbohydrate crop that is a staple food for more than 65% of the population in China. Based on habitual dietary patterns, T2DM patients in China face the challenge of adapting to carbohydrate-restricted diets, especially at breakfast. Therefore, we attempted to replace traditional rice soup with Yam Gruel to lower carbohydrate intake.

Yam Gruel is a classic diet therapy prescription from Xichun Zhang, a famous doctor of traditional Chinese medicine in the Ming Dynasty [29]. Yam Gruel contains exclusively Chinese yam, which is not only a common food in China with a long history but also an herb that is typically used to invigorate the spleen and stomach to regulate metabolism. In China, Yam Gruel is recommended as part of a healthy diet for T2DM patients. In accordance with the findings of previous investigations, dietary intervention combined with Yam Gruel can reduce fasting blood glucose (FBG) levels and 2-h postprandial blood glucose (2hPBG) levels as well as improve the blood lipid profile (namely, TG, TC, and LDL-C levels) in T2DM patients; these findings are consistent with the results of other animal experiments [30, 31]. To some extent, the combination of Yam Gruel with metformin is more effective than either of the other agents alone and has advantages in improving insulin sensitivity and decreasing FBG and TG levels [32, 33]. AMPK is a key regulatory molecule that ameliorates hepatic insulin resistance and lipid metabolism. Many studies have shown that AMPK/ACC/CPT-1 A pathway activation in the liver stimulates the oxidation of free fatty acids to decrease lipid accumulation [34, 35]. Previous studies have proven that Yam Gruel can prevent insulin resistance and improve glucose metabolism via activation of the AMPK pathway in skeletal muscles and pancreases of rats with T2DM [32, 33]. However, whether Yam Gruel can affect hepatic lipid metabolism through the AMPK/ACC/CPT-1 A pathway remains unknown.

Given that Yam Gruel potentially stimulates the AMPK pathway in skeletal muscles and the pancreas, together with its significant association with lipid metabolism, this study fed diabetic rats with Yam Gruel to evaluate its impact on serum lipid levels, liver function, and hepatic steatosis. Additionally, AMPK pathway activation in liver tissues was assessed by q-PCR and western blotting. The findings of the present study reveal crucial insights into the mechanism by which Yam Gruel exerts its anti-lipid effects on diabetic rats.

## Methodology

### Yam Gruel preparation

Yam Gruel was produced following the methodologies outlined in the publication “Records of Traditional Chinese and Western Medicine in Combination” by Xichun Zhang [36]. During preparation, the skin was removed from 150 g of raw Chinese yam, and then, the yams were sliced; each Chinese yam slice weighed 125 g. Each Chinese yam slice was added to a homogenizer with 50 mL of water to form a thick paste. The paste was placed in 250 mL of cold water and then heated under a low to medium heat setting for a duration of 30 s each time. The process of boiling was conducted three times at 30-s intervals. Throughout the boiling phase, the paste was carefully mixed. The final concentration of Yam Gruel was approximately 0.5 g/mL [31, 37].

### Animals and the T2DM rat model

Forty SPF-grade male Wistar rats (8 weeks old, 200 ± 20 g) were procured from Shanghai SLAC Laboratory Animal Co., Ltd. (Shanghai, China). Following a 1-week period of adaptive breeding, 40 rats were randomly divided into two separate groups; for six weeks, the control group (*n* = 6) was provided basic food, and the T2DM group (*n* = 34) was provided a fat-rich diet (15% sucrose, 10% lard, 10% yolk powder, 4% cholesterol, 60.7% standard diet and 0.3% chocolate). After being fed a high-fat diet for 6 weeks followed by 12 h of fasting, the rats in the T2DM model group received an intraperitoneal injection of 25 mg/kg + m^2^ streptozotocin [STZ, freshly prepared in citrate buffer solution (pH 4.5)], and another intraperitoneal injection of STZ was administered after a 72-h interval. Moreover, the rats in the control group were administered a comparable volume (25 mg/kg + m^2^) of citrate buffer. Fasting blood glucose levels were assessed on the third and seventh days after injection by extracting blood from the tail vein and utilizing a consistent, portable blood glucose meter. If the postfasting blood glucose level of a rat in the model group was greater than 11.1 mmol/L, the model was considered to have been successfully established. Eventually, 24 successful T2DM model rats and 6 control rats were chosen for intervention in subsequent studies. The Laboratory Animal Ethics Committee of Fujian University of Traditional Chinese Medicine approved the present study (FJTCM IACUC 2019056).

### Animal regrouping and interventions

**Fig. 1:**
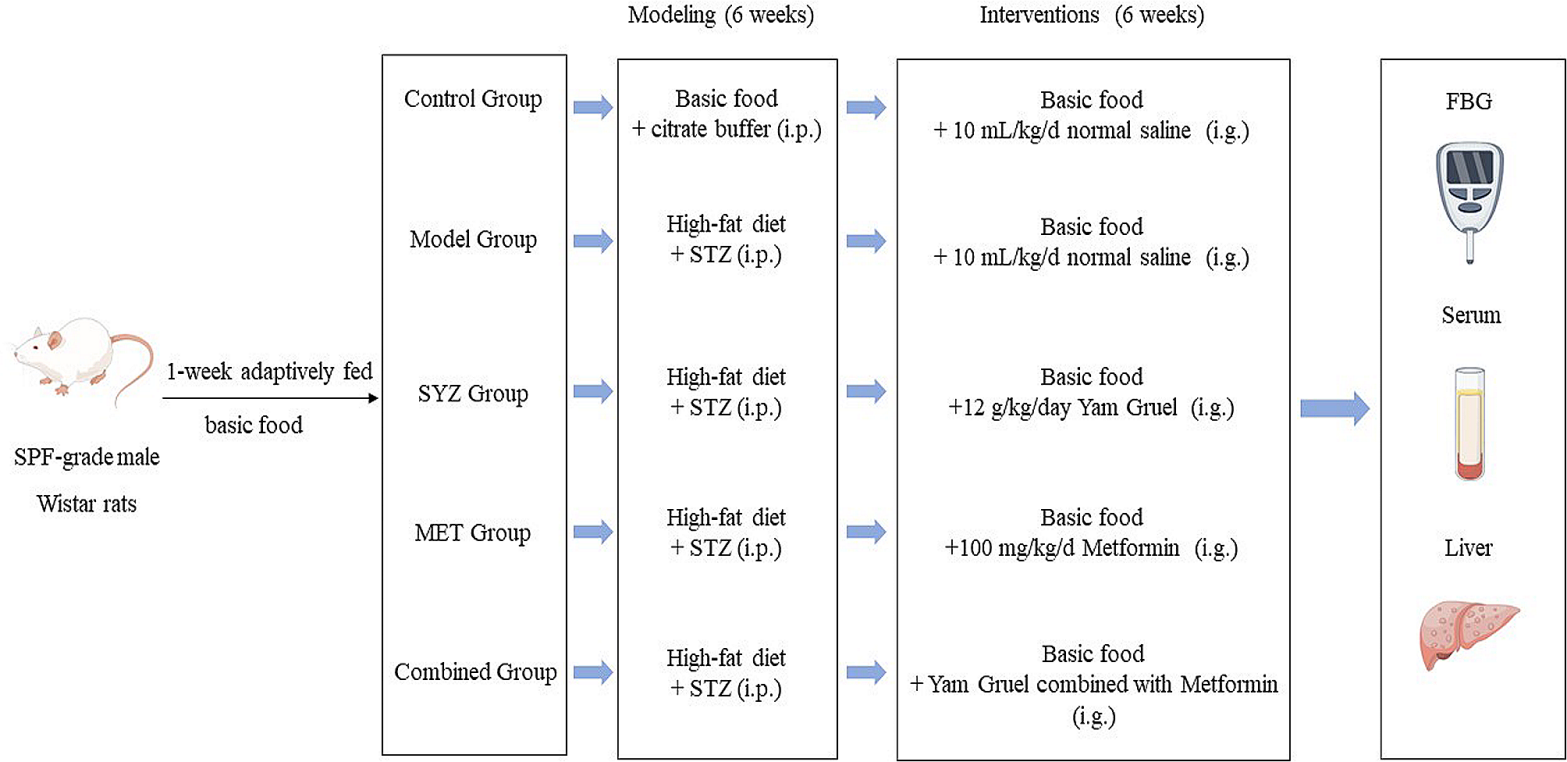
The flowchart of animal modelling and diet/drug administration treatment

A total of thirty rats were randomly allocated to five different groups: (1) the control group (normal saline, 10 mL/kg/d, *n* = 6); (2) the model group (normal saline, 10 mL/kg/d, *n* = 6); (3) the SYZ group (Yam Gruel, 12 g/kg/day, *n* = 6); (4) the MET group (metformin, 100 mg/kg/d, *n* = 6); and (5) the Combined group (Yam Gruel combined with metformin, the gavage dose was the same as described above, *n* = 6). Each treatment lasted for six weeks. Throughout the trial, the body weights of each group of rats were evaluated on a weekly basis to determine the optimal gavage dose. The flowchart of animal modelling and diet/drug administration treatment is shown in Fig. 1.

### Tissue processing

After 6 weeks of treatment, the rats were subjected to anaesthesia using a 2% solution of sodium pentobarbital (45 mg/kg) after 12 h of overnight fasting. Blood was drawn from the abdominal aorta and transferred to BD Vacutainer® Venous Blood Collection Tubes (SST™ Serum Separation Tubes Becton, Dickinson and Company, Franklin Lakes, NJ, USA), and then, the samples were centrifuged to extract the serum (3000 rpm, 10 min). The serum was frozen at − 80 °C for future processing. Complete liver tissues were removed, weighed, and cut into smaller pieces. Two pieces of liver tissue per sample were preserved at − 80 °C for western blotting and q-PCR, while the two other pieces per sample were immediately frozen in liquid nitrogen for Oil Red O staining.

### Serum biochemical analysis

Serum levels of TC, TG, HDL-C, LDL-C, nonesterified fatty acids (NEFA), alanine aminotransferase (ALT), and aspartic acid transaminase (AST) were measured following the instructions of the respective test kits (NEFA Colorimetric Assay Kit, Elabscience, Wuhan, China; the serum TC, TG, HDL-C, LDL-C, ALT and AST biochemical analysis kits, Rayto, Shenzhen, China). Briefly, all the serum samples were thawed and subjected to centrifugation for 10 min at 4 °C and 3000 rpm. A 15-µL aliquot of each serum sample was added to a Chemray800 Automatic Biochemistry Analyzer to measure the lipid profile and liver function.

### Oil Red O staining of liver tissues

To evaluate hepatic steatosis, 8-µm-thick frozen liver slices were generated and subjected to Oil Red O staining. Afterwards, 0.5 g of Oil Red O (Sigma‒Aldrich, St. Louis, MO, USA) was dissolved in 100 mL of 98% isopropanol to create a saturated Oil Red O stain. The Oil Red O stain that was suitable for use was produced through dilution, whereby a saturated Oil red O solution was diluted at a ratio of 6:4 with distilled water and then filtered after standing for 10 min. Staining was performed in the dark at room temperature for 15 min. The tissue slices were placed in 60% ethanol to continue colour separation [38]. Images were captured using a fluorescence microscope (Leica inverted fluorescence microscope DM IL LED, Leica, Wetzlar, Germany). Image-Pro Plus 6.0 software was used to determine the positive staining area and the number of lipid droplets.

### Extraction of proteins and western blotting

Total protein was extracted from liver tissues from rats in each group using RIPA lysis buffer (MA0151; Meilunbio®, Dalian, China) at 4 °C. The proteins were resolved via SDS‒PAGE and subsequently transferred to PVDF membranes. TBST containing 5% skim milk was used to block the membranes at 37 °C for 1 h. Then, the membranes were incubated at 4 °C overnight with the indicated antibodies and subsequently with the appropriate HRP-conjugated secondary antibodies (1:1000 dilution) at room temperature for 1 h [39]. The primary antibodies that were used were an AMPK and ACC Antibody Sampler Kit (#9957 Cell Signaling Technology, Shanghai, China), a DCMC rabbit pAb (YN4175, ImmunoWay Biotechnology Company, Texas, USA), a CPT1A rabbit pAb (YN3388, ImmunoWay Biotechnology Company, Texas, USA), and an anti-beta-actin mouse mAb (GB12001, Wuhan Servicebio Technology Co., Ltd., Wuhan, China). Using enhanced chemiluminescence techniques, immunoreactive proteins were identified. Grayscale analysis was then performed to quantify protein expression using ImageJ software (version 6.0.0.260; Media Cybernetics Corporation, USA).

### Real-time PCR analysis

Total RNA was extracted using RNA Reagent (TIANGEN BIOTECH [BEIJING] Co., Ltd., Beijing, China) according to the manufacturer’s instructions. Reverse transcription was performed according to the handbook of NovoScript® 1st Strand cDNA Synthesis SuperMix (gDNA Purge) (Novoprotein Scientific, Inc., Beijing, China). The expression levels of protein kinase AMP-activated catalytic subunit alpha 2 (Prkaa2), acetyl-CoA carboxylase alpha (Acaca), malonyl-CoA decarboxylase (MLYCD), carnitine palmitoyltransferase 1 A (CPT1A), and actin beta (Actb) were quantified using an ABI Step One Plus Real-Time PCR system (Thermo Fisher Scientific, Inc., Massachusetts, USA) and NovoStart® SYBR qPCR Supermix Plus (Novoprotein Scientific, Inc., China). The Prkaa2, Acaca, MLYCD, and CPT1A mRNA expression levels in each sample were normalized to the level of Actb. Table 1 shows the primers that were used to amplify these genes. Each experiment was conducted in duplicate. The cycling procedure conditions were as follows: 10 min at 95 °C, followed by 40 cycles of 15 s at 95 °C and a minute at 60 °C. The data obtained were analysed using the comparative Ct technique (2^−ΔΔCt^ method), where ΔΔCt = ΔCt sample – ΔCt.

**Table 1:** Primer sequences

Gene name	Forward primer	Reverse primer	Length(bp)
Prkaa2	TCCTTCATGGACGATATGGCC	ATCCAGTGGACAGCGTGCTTT	108
Acaca	GCGGCTCTGGAGGTATATGT	TTAGCGTGGGGATGTTCCCT	151
MLYCD	CAGGGAAAGGAGTATGGGAGG	TTCTCAGACTTCGCCCACTCA	134
CPT1A	CTTCCCCTTACTGGTTCC	ACTCTCCCGCTGTTGTCC	202
Actb	ACTCTGTGTGGATTGGTGGC	AGCTCAGTAACAGTCCGCCT	137

### Statistical analysis

The data were analysed utilizing SPSS 22.0 software (IBM Corp., Armonk, NY, USA) and are presented as the mean ± standard deviation (SD). Before conducting the test, normality tests were performed. One-way analysis of variance (ANOVA) was used to assess the statistical significance of differences among multiple groups, and the LSD test (assuming equal variances) and Tamhane’s T2 test (without the assumption of equal variances) were used. For comparisons of multiple time points between groups, a repeated-measures ANOVA was utilized. A *P* value less than 0.05 was considered to indicate statistical significance.

## Results

### Influence of Yam Gruel on body weight and FBG levels in T2DM model rats

**Fig. 2:**
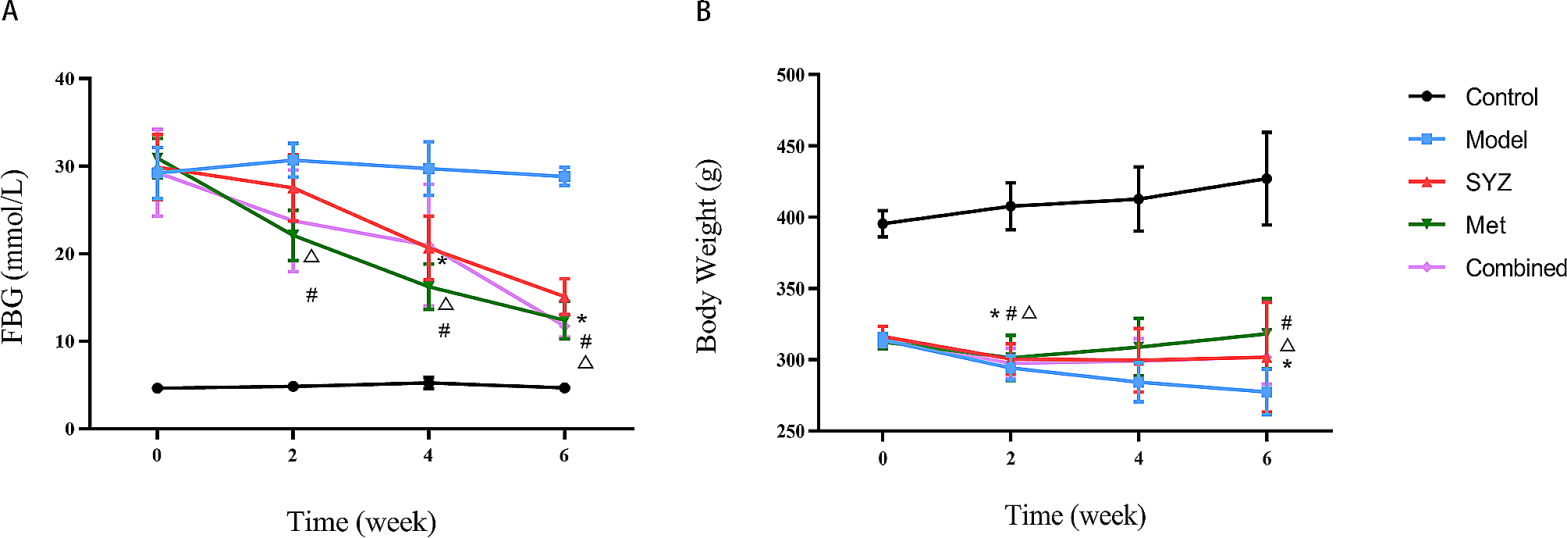
Impact of Yam Gruel on body weight and FBG within T2DM rats. **(A)** the changes of FBG of rats within all groups throughout the 6-week intervention; **(B)** rat’s body weight changes within all groups throughout the 6-week intervention. The data are expressed as mean ± SD (*N* = 6). ^*^P < 0.05, contrasted to the previous time point in the SYZ group; ^#^*P* < 0.05, contrasted to the previous time point in the MET group; ^Δ^*P* < 0.05, contrasted to the previous time point in the Combined group

Fig. 2 shows that rats with diabetes exhibited significant decreases in body weight and increases in FBG levels after the model was established (*P* < 0.01), which indicated the successful establishment of the T2DM models. Throughout the entire experimental phase, the body weight of rats in the model group continued to decrease (*P* < 0.05). The results of the intragroup analysis showed that the weights of the diabetic rats in the SYZ, MET and Combined groups still decreased until the second week of intervention (*P* < 0.05); this trend began to plateau beginning the fourth week (*P* > 0.05) and finally was even reversed (*P* < 0.05). Compared to those in the model group, the FBG concentrations in the SYZ, MET and Combined groups decreased beginning in the second week and were significantly lower in the fourth and sixth weeks after treatment (*P* < 0.01). There was no statistically significant difference in the FBG levels between the control group and the model group at different time points (*P* > 0.05). After four and six weeks of intervention, the FBG levels of the rats in the SYZ group were markedly lower than those before the intervention (*P* < 0.05). Similarly, the FBG levels of rats in both the MET and Combined groups were significantly lower at the second, fourth, and sixth weeks of intervention than at the previous time points (*P* < 0.05).

### Yam Gruel reduced liver weight and index in T2DM model rats

As shown in Table 2, the liver weights and liver-to-body weight ratio of the T2DM model rats in the model group were significantly greater (*P* < 0.01) than those in the control group. However, in contrast to those in the model group, the weight and liver indices of the rats with T2DM in the SYZ, MET, and Combined groups were significantly lower (*P* < 0.01) after six weeks of treatment by gavage. According to pairwise comparisons, no statistically significant differences were found in liver weight or indices among the SYZ, MET, or combined groups (*P* > 0.05).

**Table 2:** Impact of Yam Gruel on liver weight and index in T2DM rats

Group	Body weight (g)	Liver weight (g)	Liver index	
Control	427.00 ± 32.58	11.21 ± 0.71	2.63 ± 0.16	
Model	277.33 ± 16.02	15.51 ± 1.28^**^	5.32 ± 0.42^**^	
SYZ	301.63 ± 38.66	10.03 ± 0.35^##^	3.37 ± 0.42^*##^	
MET	318.13 ± 24.69	10.42 ± 0.78^##^	3.28 ± 0.16^**##^	
Combine	301.86 ± 19.30	10.04 ± 0.60^##^	3.33 ± 0.18^**##^	

The data are expressed as mean ± SD (*N* = 6). ^*^P < 0.05, ^**^P < 0.01, compared with the control group; ^#^*P* < 0.05, ^##^*P* < 0.01, compared with the model group

### Yam Gruel reduced the lipid profile and improved liver function in T2DM model rats

**Fig. 3:**
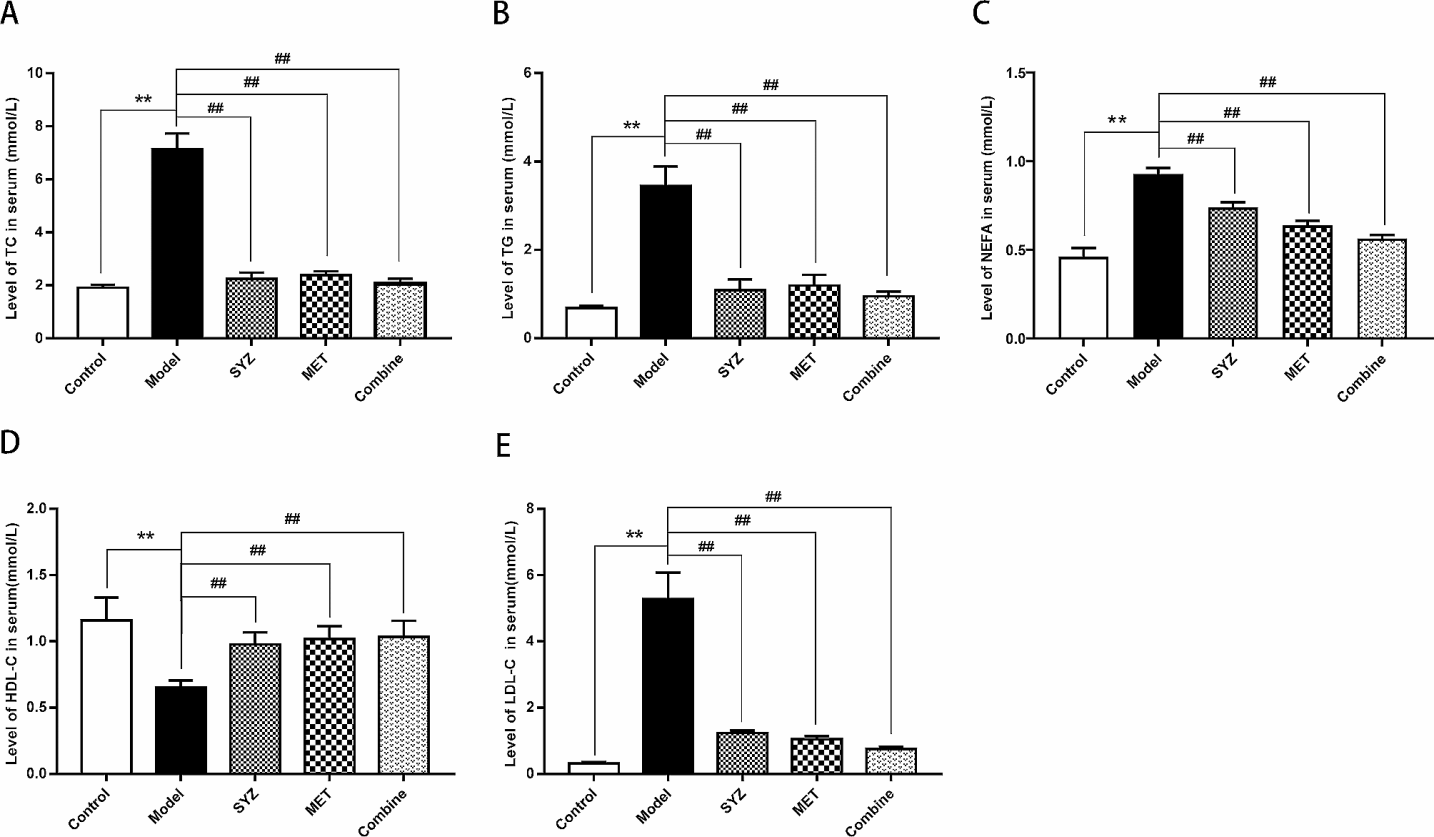
Yam Gruel decreased the lipid profile levels in the serum of diabetic rats by serum biochemical analysis and Elisa. **(A)** the level of total cholesterol (TC) in the rats across all groups; **(B)** the level of triglyceride (TG) in the rats across all groups; **(C)** the level of nonesterified fatty acids (NFFA) in the serum of rats across all groups; **(D)** HDL-C in the serum of rats across all groups; **(E)** LDL-C level within the serum of rats in each group. The data are expressed as mean ± SD (*N* = 6). ^**^P < 0.01, contrasted to the control group; ^##^*P* < 0.01, contrasted to model group

**Fig. 4:**
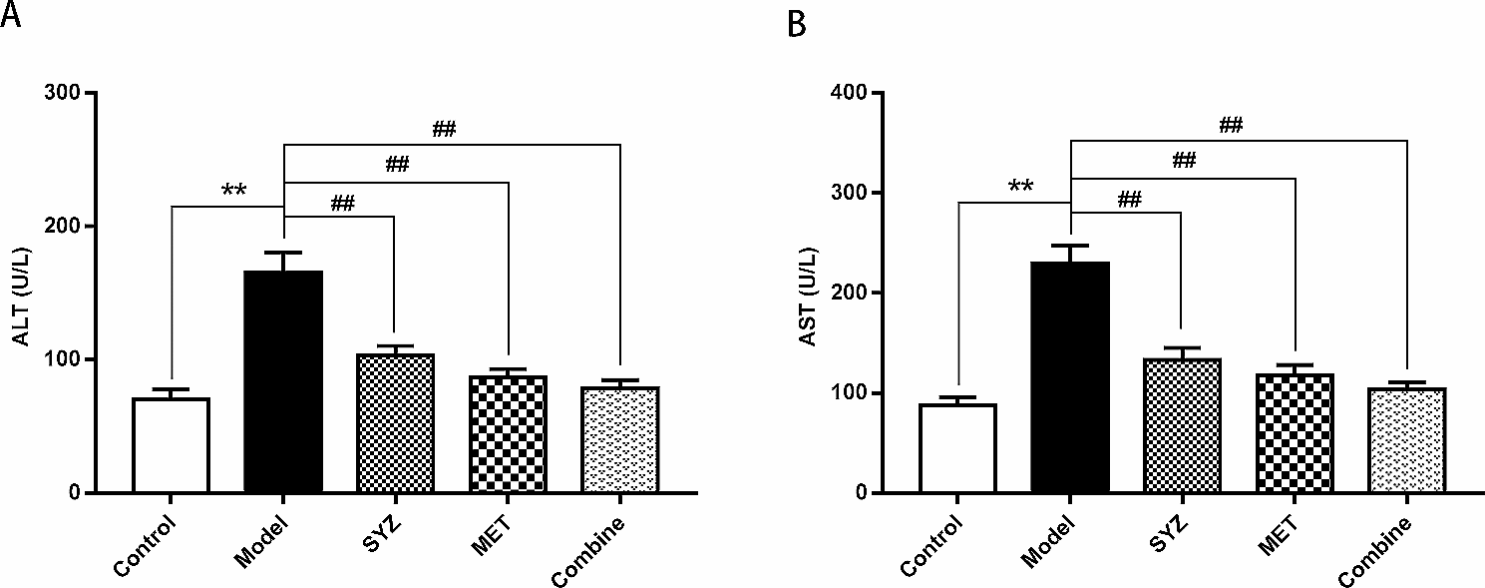
Yam Gruel improved liver function in the serum of diabetic rats by serum biochemical analysis. **(A)** Alanine aminotransferase (ALT) within rat’s serum across all groups; **(B)** aspartic acid transaminase (AST) in the serum of rats in each group. The data was reported as mean ± SD (*N* = 6). ^**^P < 0.01, contrasted to control group; ^##^*P* < 0.01, contrasted to model group

After six weeks of treatment, the lipid profiles, including serum levels of HDL-C, LDL-C, TC, TG and NFFA, were measured by biochemical analysis and Elisa. As shown in Fig. 3, the serum levels of LDL-C, TC, TG and NEFA in the model group were significantly greater than those in the control group, whereas a significant decrease was observed in the serum HDL-C levels in the model group (*P* < 0.01). In contrast to those in the model group, the serum concentrations of LDL-C, TG, TC and NEFA were significantly decreased, whereas the serum HDL-C level exhibited a notable increase in all three treatment groups (*P* < 0.01). As shown in the results of the biochemical analysis in Fig. 4, liver function was examined by measuring the serum AST and ALT levels. The serum ALT and AST concentrations were increased relative to those in the control group (*P* < 0.01). In contrast to those in the model group, the serum ALT and AST concentrations were lower in the SYZ, MET, and Combined groups (*P* < 0.01). Moreover, pairwise comparisons among the three therapy groups (SYZ, MET, and Combined) showed that the decreases in the TC, LDL-C, NEFA, ALT, and AST serum levels in the combination group were substantially greater than those in the SYZ or MET groups (*P* < 0.05, *P* < 0.01, respectively). These results showed that Yam Gruel treatment may enhance serum lipid metabolism and liver function.

### Yam Gruel enhanced liver fat accumulation and pathological changes

First, the macromorphologies of liver tissues from each group were examined after six weeks of treatment. Macroscopically, the livers in the control group had a healthy reddish-brown colour with a soft and elastic appearance and a sharp edge, smooth surface, and normal size. Concurrently, the livers in the model group displayed obvious enlargement and numerous areas of white fat accumulation. Compared to those in the model group, liver size and fat accumulation were substantially lower in all three treatment groups.

**Fig. 5:**
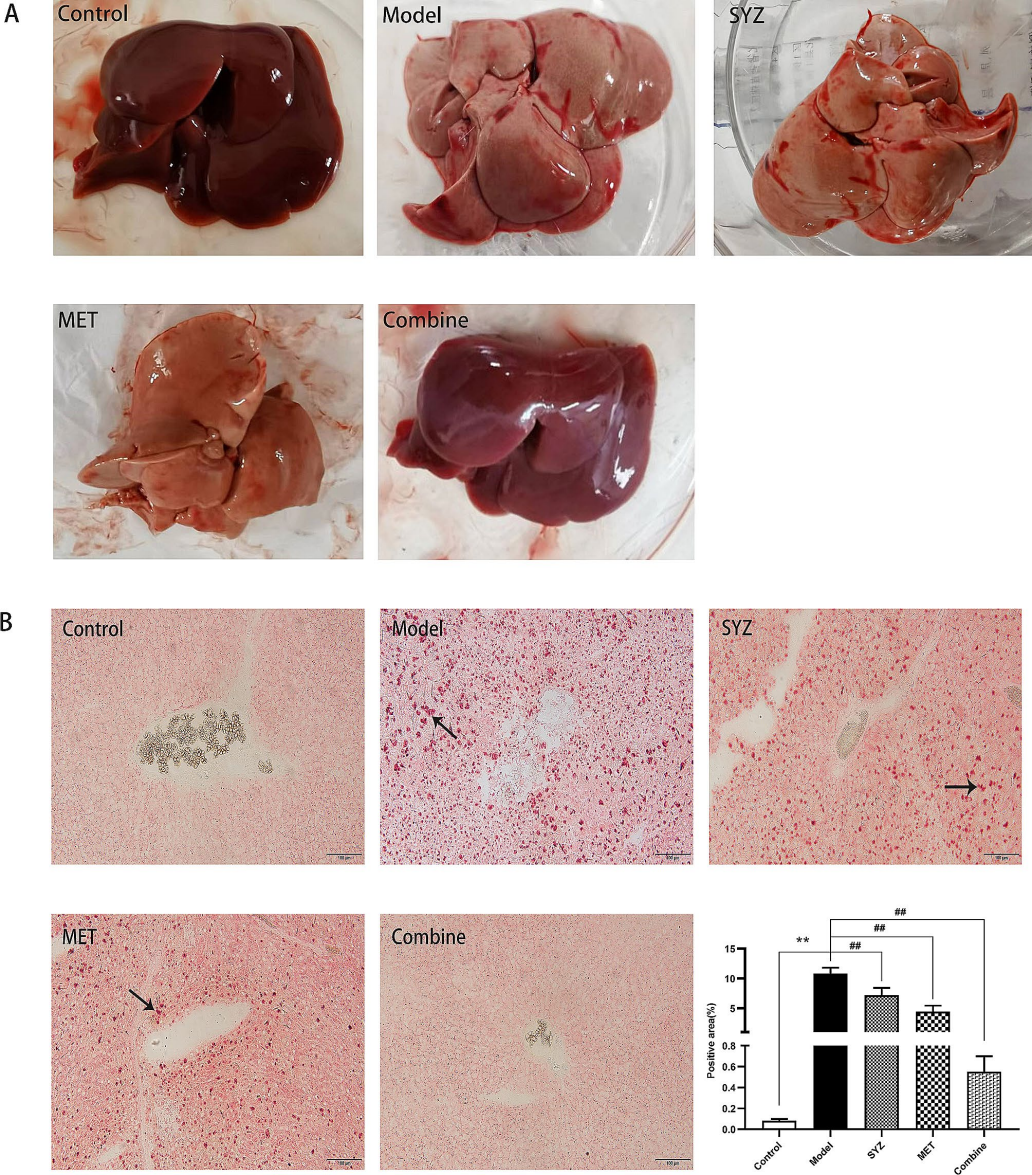
The macroscopic and histopathological alterations of liver tissues in diabetic rats by Red Oil O staining after 6-week interventions. **(A)** The macroscopic liver appearance of rats in each group; **(B)** The histopathological alterations of liver tissues of rats in each group. Black arrows in Red Oil O staining indicate bigger red lipid droplets. Microscopic images with 200 × total magnification. Scale bar: 100 μm. The data was presented as mean ± SD (*N* = 6)

Then, histopathological changes in the liver tissues of all the groups were assessed. As shown in Fig. 5, staining with Oil Red O revealed that Yam Gruel, metformin, and the combination treatment visibly attenuated the accumulation of hepatic lipids, as shown by fewer ballooning hepatocytes and intracellular lipid droplets. In contrast to that in the control group, the area of positive Oil Red O staining in the model group was markedly increased (*P* < 0.01), whereas the area of positive staining in the SYZ, MET, and Combined groups was significantly lower than that in the model group (*P* < 0.01). The area of positive staining in the Combined group was significantly lower than that in the SYZ group and the MET group (*P* < 0.01).

### Yam Gruel activated the AMPK/ACC/CPT-1 pathway in liver tissues

**Fig. 6:**
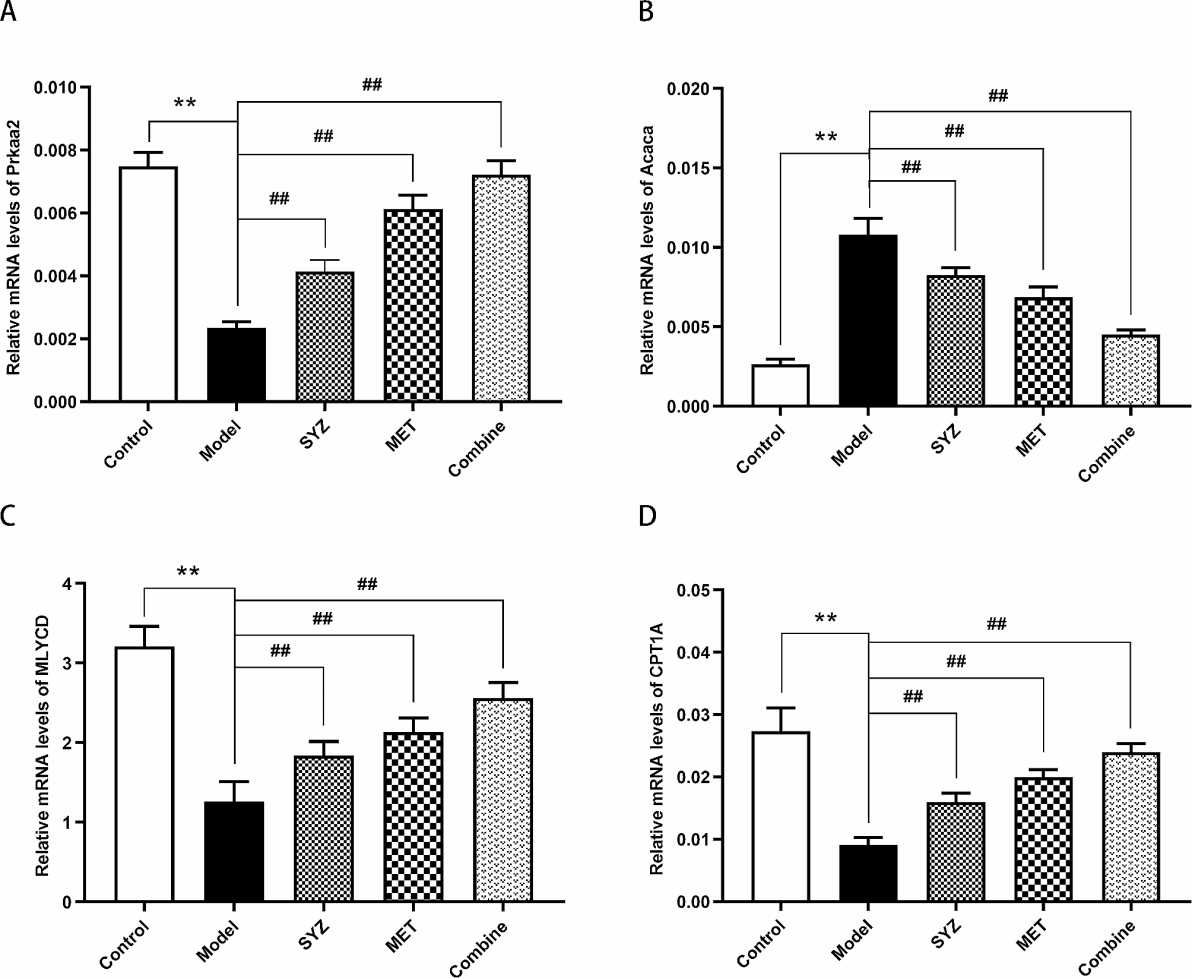
Impact of Yam Gruel on the mRNA expression of AMPK/ACC/CPT-1 pathway by RT-PCR analysis in liver tissue of T2DM rats. **(A)** The relative mRNA level of Prkaa2; **(B)** the relative mRNA level of Acaca; **(C)** the relative mRNA level of MLYCD; **(D)** the relative mRNA level of CPT1A. The data was reported as mean ± SD (*N* = 6). **P < 0.01, contrasted to control group; ^##^*P* < 0.01, contrasted to model group

To investigate the potential pathway by which Yam Gruel improves hepatic lipid metabolism in rats with T2DM, we investigated the activation of the AMPK/ACC/CPT-1 pathway. Compared with those in the control group, the relative mRNA levels of Prkaa2, MLYCD, and CPT1A in the liver tissues of diabetic rats were significantly decreased; however, the mRNA expression level of Acaca was significantly greater (*P* < 0.01). However, treatment with Yam Gruel and/or metformin for six weeks substantially reversed the decreases in the mRNA expression of Prkaa2, MLYCD, and CPT1A and the increases in the mRNA expression of Acaca (*P* < 0.01), as shown in Fig. 6A–D. A significant difference was observed in the relative mRNA levels of Prkaa2, MLYCD, Acaca and CPT1A between the combined group and the Yam Gruel alone and metformin alone groups (*P* < 0.05, *P* < 0.01).

**Fig. 7:**
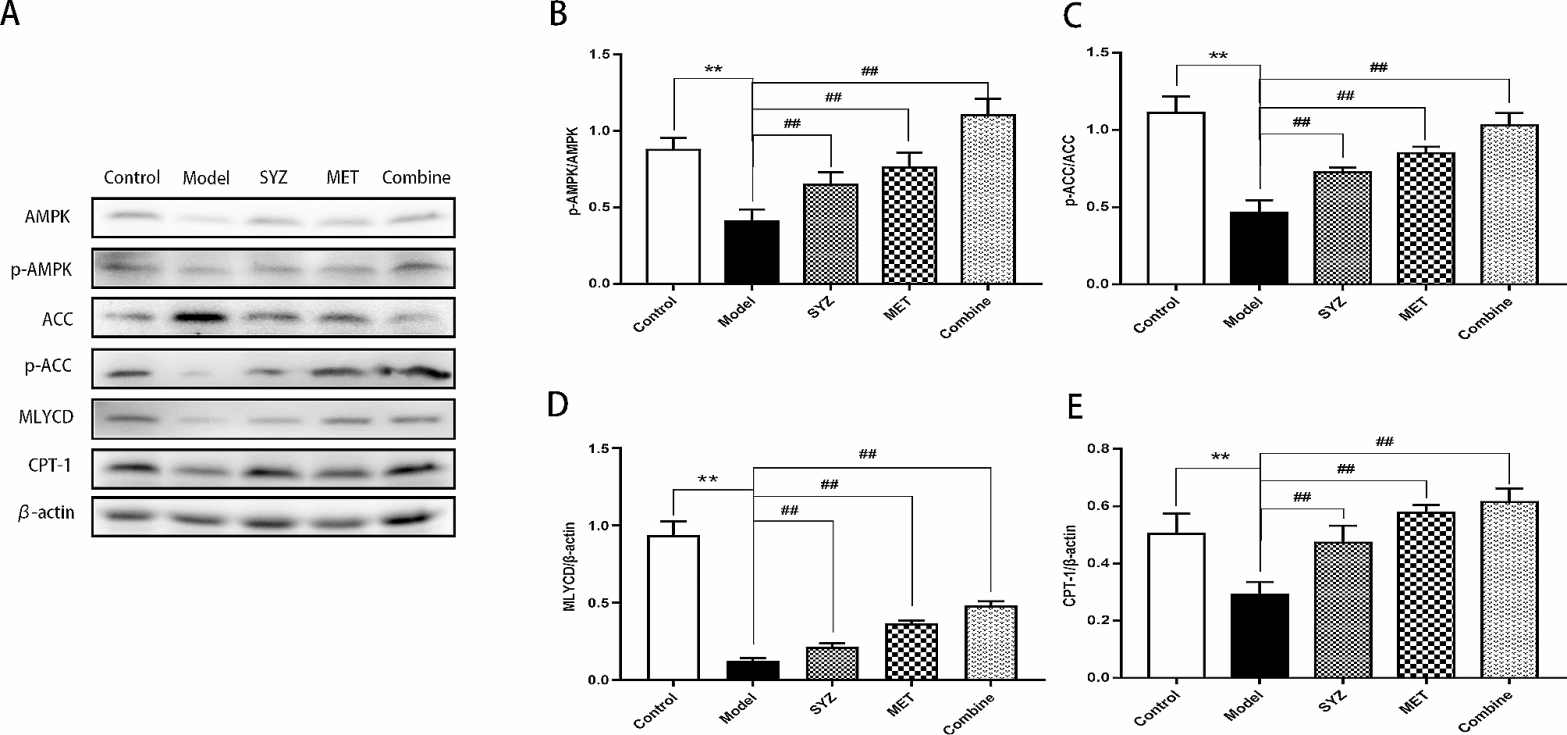
Impact of Yam Gruel on protein expression of AMPK/ACC/CPT-1 pathway by Western blot analysis within T2DM rats’ liver tissues. **(A)** The images of protein from the AMPK/ACC/CPT-1 pathway by Western blot; **(B)** the protein expression level of p-AMPK/AMPK; **(C)** the protein level of p-ACC/ACC; **(D)** the protein level of MCoA/β-actin; **(E)** the protein level of CPT-1/β-actin. The data was presented as mean ± SD. (*N* = 6). **P < 0.01, contrasted to control group; ^##^*P* < 0.01, contrasted to model group

As shown in Fig. 7A–E, the protein levels of p-ACC/ACC, p-AMPK/AMPK, MLYCD, and CPT-1 were significantly lower (*P* < 0.01) in the livers of diabetic rats in the model group than in those of the control group. In contrast, protein expression levels in rat liver tissues were significantly higher in the three treatment groups than in the control group (*P* < 0.01 for all). Moreover, the protein levels of p-ACC/ACC, p-AMPK/AMPK and MLYCD were significantly higher in the combined intervention group than in the single intervention group (*P* < 0.01). This trend was also observed for CPT-1 between the SYZ group and the Combined group (*P* < 0.01), but was not observed between the MET group and the Combined group (*P* > 0.05).

## Discussion

According to the principles of traditional Chinese medicine, dyslipidaemia in T2DM patients is mainly caused by dysfunction of the spleen, which can enhance the absorption and transportation of nutrient substances. In recent years, the “Homogeny of the Spleen and Pancreas” theory proposed by He Shaoqi (a famous doctor of traditional Chinese medicine [TCM]) [36] supports the view that spleen-qi deficiency disrupts glucose and lipid metabolism. Many studies have confirmed that spleen Qi and Yin deficiency is the most common TCM syndrome among T2DM patients with hyperlipidaemia [40]. Therefore, strengthening spleen Qi and nourishing Yin are important principles for the clinical treatment of abnormal glucose-lipid metabolism caused by T2DM. A previous meta-analysis showed that traditional Chinese dietary therapy that involves supplementing Qi and nourishing Yin could lower the levels of FBG, 2hPBG, and HbA1c in T2DM patients [37]. Several experimental animal studies have revealed that the “supplementing Qi and nourishing Yin” prescription can significantly reduce the liver indices, blood lipids, and extent of steatosis in liver tissues of rats with diabetes that was induced by a combination of high-fat diet and STZ [30, 41, 42].

In the present study, Yam Gruel and metformin were selected as interventions that were used and compared in the treatment of T2DM model rats. Yam Gruel is mainly composed of Chinese yam, which is a famous traditional food and medicine in China. Chinese yams are widely distributed in China, but Jiaozuo in Henan Province has become the undisputable area of Chinese yam production due to the high quality of Chinese yams that are produced in this region. Consequently, the Chinese yam used in the study was produced by Jiaozuo. Chinese yams are classified as “Qi-Invigorating Herbs”, and their main function is to strengthen spleen qi and nourish spleen yin, which can effectively improve lipid and glucose metabolism and are among the core medicines that are used in the treatment of T2DM [43]. In modern pharmacology, the beneficial effects of Chinese yams depend on their active ingredients. In recent years, several in vitro and in vivo investigations have shown that the major bioactive ingredients that are isolated from Chinese yams, such as polysaccharides, diosgenin, polyphenols, allantoin, and starch, ameliorate glucose and lipid metabolism disorders in T2DM patients [44–46]. Metformin is the first choice for T2DM treatment according to the Chinese guidelines for the prevention and treatment [3]. Metformin, which is a potent activator of AMPK, can inhibit hepatic glucose synthesis, improve lipid metabolism, and lower blood glucose levels [47].

Throughout the current investigation, compared with those in the control group, the FBG levels and serum lipid concentrations (including TG, TC, LDL-C, and NEFA concentration) in the diabetic rats markedly increased. These findings indicate that T2DM model rats exhibit obvious disorders of glucose and lipid metabolism. Feeding diabetic rats Yam Gruel not only resulted in a substantial reduction in FBG levels from the fourth to sixth week of treatment but also resulted in a notable decrease in TC, TG, LDL-C, and NEFA levels, which are important biochemical indicators of lipid metabolism. Expert consensus indicates that a decrease in LDL-C levels is the fundamental objective of blood lipid regulation in patients who are diagnosed with type 2 diabetes [14, 48]. After the 6-week intervention, all three treatments lowered serum LDL-C levels by more than 50%. Because the role of the liver in regulating lipids depends on its structure and function, liver function and pathological changes were evaluated. The outcomes showed that the ALT and AST levels in diabetic rats were elevated relative to those in the control group. Concurrently, livers from the model group displayed obvious enlargement and numerous areas of fat accumulation. These findings showed that diabetic rats had liver metabolic dysfunction and hepatocyte steatosis. After up to 6 weeks of treatment by gavage, ALT and AST levels were significantly lower, and the area of positive Oil Red O staining was significantly lower in the SYZ, MET, and Combined groups. In brief, Yam Gruel and/or metformin exert liver-protective effects and could constitute the important diet therapy for alleviating T2DM-related hyperlipidaemia. The utilization of a combination approach demonstrated superior efficacy in regulating FBG levels, liver weight and liver indices; improving serum lipid metabolism (TC, LDL-C, and NEFA levels); improving liver function; and mitigating liver steatosis compared to individual interventions. This outcome strongly suggested that the synergistic effect of the combined diet and drug intervention played a significant role in achieving these positive results.

However, there has been no uniform conclusion on the mechanism of anti-diabetic and lipid metabolism-improving actions of Yam Gruel thus far. Glucose-lipid homeostasis is most strongly influenced by the liver, which is the centre of energetic changes in the body and contributes to the synthesis of fatty acids and cholesterol and the oxidation of fatty acids. The ability of the liver to regulate lipid metabolism is dependent not only on normal tissue structure and function but also on multiple cellular signalling pathways. AMP-activated protein kinase (AMPK), which contains one catalytic subunit (α1 or α2) and two regulatory subunits, is considered a potential target for maintaining energy homeostasis in liver, muscle, and adipose tissues [49]. AMPK is particularly closely associated with fatty acid oxidation and hepatic lipogenesis [50]. AMPK dephosphorylation causes the sequential dephosphorylation of ACC, increasing malonyl-CoA levels and inhibiting CPT1A-dependent fatty acid oxidation. Malonyl-CoA decarboxylase (MLYCD), which generates acetyl-CoA from malonyl-CoA, is activated by AMPK [51, 52]. Xu et al. revealed that dioscin decreased the levels of ACC gene expression in rats with T2DM that was induced with high-fat diet and STZ, leading to a decrease in lipogenesis [53]. In a study on rats with STZ-induced diabetes, scientists demonstrated that *Callicarpa nudiflora* extract might enhance oral glucose tolerance, lipid metabolism, and insulin resistance and ameliorate diabetes-associated liver and pancreatic impairment by activating the AMPK-ACC pathway [54]. In the present study, the protein levels of p-ACC/ACC, p-AMPK/AMPK, MLYCD, and CPT-1 were significantly lower in the livers of diabetic rats in the model group than those in the control group. These results demonstrated that AMPK phosphorylation was attenuated and the liver-specific AMPK pathway was suppressed in T2DM model rats. Administration of Yam Gruel upregulated the mRNA expression of Prkaa2, MLYCD, and CPT1A and downregulated that of Acaca. The protein levels of p-ACC/ACC, p-AMPK/AMPK, MLYCD, and CPT-1 were increased in liver tissues, which was consistent with the observed changes in gene expression. Taken together, these findings indicate that the impact of Yam Gruel on the recovery of lipid metabolism in diabetic rats may be mediated by AMPK signalling pathway activation. Notably, Yam Gruel combined with metformin showed a considerable ability to activate AMPK to suppress adipogenesis in the liver.

### Strengths and limitations

The present investigation primarily examined the issue of dietary treatment for patients with T2DM in China, with a comprehensive emphasis on the utilization of Yam Gruel as a dietary supplement to decrease carbohydrate consumption. This is the first study in which the impact of Yam Gruel on improving liver function and ameliorating pathological changes in the liver were evaluated. Furthermore, the induction of the hepatic AMPK/ACC/CPT-1 pathway suggested that Yam Gruel may serve as a potential therapeutic diet for enhancing lipid metabolism in patients with T2DM.

However, this research is not without its limitations. Although the findings of this study support the notion that Yam Gruel can enhance lipid metabolism in diabetic rats by inducing the AMPK pathway in the liver, the specific bioactive compounds and metabolic constituents that are responsible for these lipid-lowering and hepatoprotective effects remain unclear. Therefore, further comprehensive and extensive investigations are needed to shed light on these aspects in future research.

## Conclusion

**Fig. 8:**
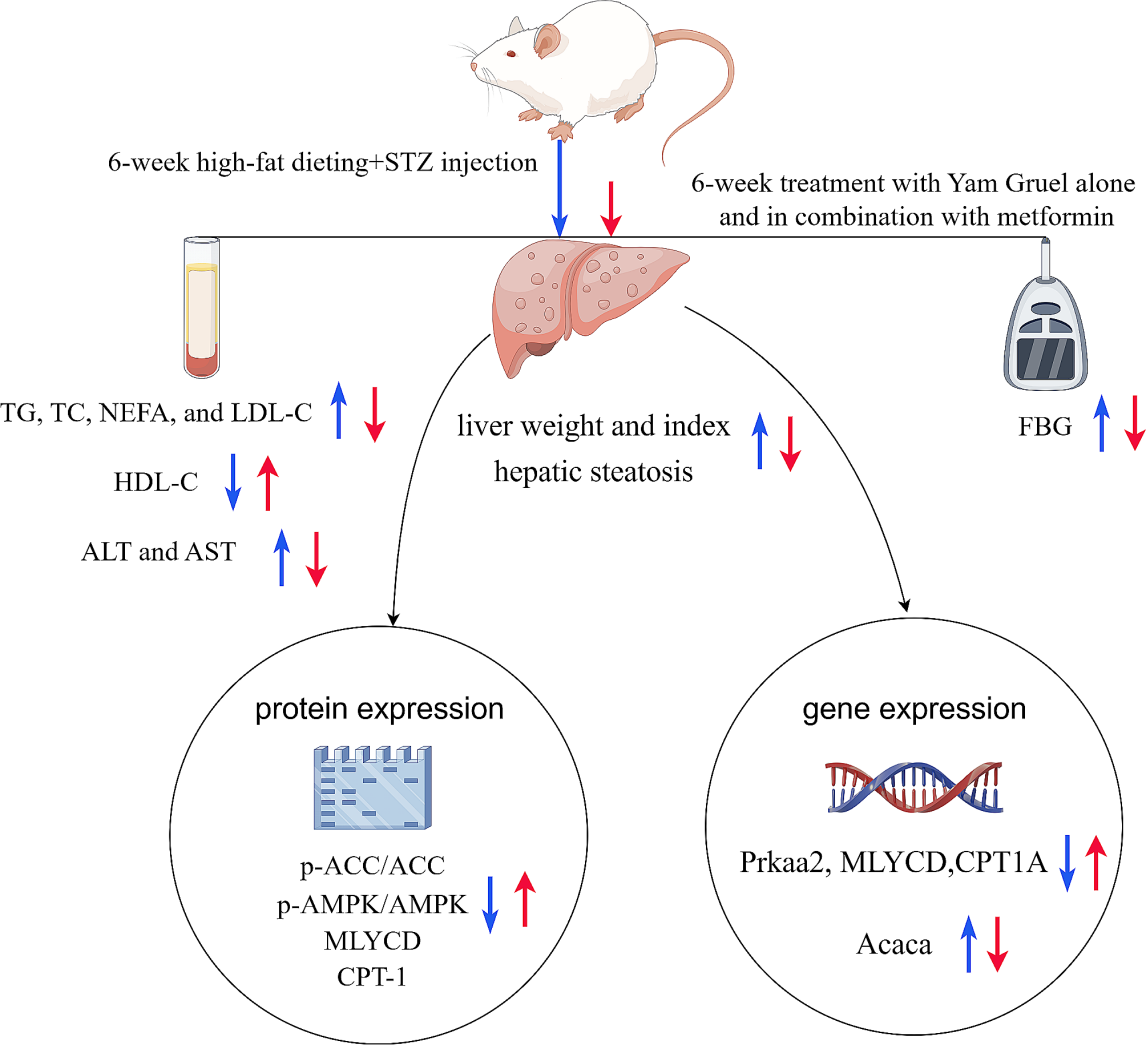
The proposed mechanism of action of Yam Gruel alone and in combination with metformin in diabetic rats. The blue arrows represent the dysfunction induced by 6 weeks high-fat dieting with STZ injection, and the red arrows represent the effects of 6 weeks of treatment with Yam Gruel alone and in combination with metformin

Yam Gruel treatment markedly reduced the liver indices and weight, the incidence of hepatic steatosis, and the accumulation of fat in rats with high-fat diet-and STZ-induced diabetes. Furthermore, combination therapy comprising Yam Gruel and metformin exerted stronger beneficial effects for relieving dyslipidaemia in T2DM, especially for controlling glycemia, treating dyslipidaemia and improving hepatic steatosis. Activation of the AMPK/ACC/CPT-1 pathway appears to be the mediator of these effects. The proposed mechanism of action of Yam Gruel alone and in combination with metformin in diabetic rats is shown in Fig. 8. The inclusion of Yam Gruel in the dietary regimen of patients with diabetes in China may effectively address the issue of excessive carbohydrate consumption while also mitigating lipid metabolism disorders and liver damage; thus, this diet could serve as a viable dietary intervention for treating dyslipidaemia associated with type 2 diabetes mellitus.

### Electronic supplementary material

Below is the link to the electronic supplementary material.

Supplementary Material 1

Supplementary Material 2

Supplementary Material 3

## Declarations

### Data and materials availability

The data pertaining to this investigation is accessible upon demand.
