# Supplementary material for: Yam Gruel alone and in combination with metformin regulates hepatic lipid metabolism disorders in a diabetic rat model by activating the AMPK/ACC/CPT-1 pathway
Source: Lipids Health Dis. 2024 Jan 25;23:28. doi: 10.1186/s12944-024-02014-2 (PMC10809441; doi:10.1186/s12944-024-02014-2)
Supplement: Supplementary file 1 — Supplementary Material 1 [file 12944_2024_2014_MOESM1_ESM.pdf]

Freescience Editorial Team

Certificate of English Editing

Paper Title

Yam Gruel regulates hepatic lipid metabolism disorders in diabetic rat model via activating the AMPK/ACC/CPT-1 pathway

Authors

Yanling Dai, Chen Qiu, Diandian Zhang, Mianli Li, Weinan Liu

This certificate is issued as a confirmation that the paper mentioned above has been proofread and edited for language clarity and grammar by professional editors of our company.

We guarantee that the original message was not distorted, and that the paper is understandable and free of errors assuming that the changes and suggestions given are accepted, and text is not altered without our knowledge.

Date of Editing: 08-21-2023
