# Supplementary material for: Yam Gruel alone and in combination with metformin regulates hepatic lipid metabolism disorders in a diabetic rat model by activating the AMPK/ACC/CPT-1 pathway
Source: Lipids Health Dis. 2024 Jan 25;23:28. doi: 10.1186/s12944-024-02014-2 (PMC10809441; doi:10.1186/s12944-024-02014-2)
Supplement: Supplementary file 2 — Supplementary Material 2 [file 12944_2024_2014_MOESM2_ESM.pdf]

No.1236 5334words 20230829 Reduction.docx

WORD COUNT 6954

TIME SUBMITTED 30-AUG-2023 03:13PM

PAPER ID 102378545

Yam Gruel regulates hepatic lipid metabolism disorders in diabetic rat model via activating the AMPK/ACC/CPT-1 pathway

Yanling Dai[1], Chen Qiu[2], Diandian Zhang[1], Mianli Li[1], Weinan Liu[3]

Correspondence to: Weinan Liu, Department of Orthopedics, People's Hospital Affiliated to Fujian University of Traditional Chinese Medicine, Fuzhou 350004, China.

Tel: +86-13799309764; Fax: 0591-83212341; E-mail: liuweinan@fjtcm.edu.cn

A comprehensive compilation of author details can be found in the concluding section of the paper

Abstract

Background As an independent and correctable risk factor, disturbances in lipid metabolism are significantly linked to type 2 diabetes mellitus (T2DM). This research investigates the mechanism underlying Yam Gruel's anti-lipid effect on diabetic rats. **Methods** First, a normal diet was given to the control group, while the diabetic rat model was created by subjecting them to a diet rich in both fat and sugar for six weeks as well as injecting them intraperitoneally with streptozotocin (STZ). After establishing the model, the rats have been separated into five distinct groups: control group, model control group, Yam Gruel (SYZ) group, Metformin (MET) group, and Combined group. The interventions within each group lasted for six weeks. The body and liver weights and index of the rats have been determined. Total cholesterol (TC), Triglyceride (TG),

high-density lipoprotein cholesterol (HDL-C), low-density lipoprotein cholesterol (LDL-C), aspartic acid transaminase (AST), high-density lipoprotein cholesterol (HDL-C), and non-esterified fatty acids (NEFA) levels were assessed. Oil Red O staining was employed for the assessment of hepatic steatosis. In addition, the levels of phosphorylation-acetyl coenzyme A carboxylase (p-ACC), acetyl coenzyme A carboxylase (ACC), AMP-activated protein kinase (AMPK), phosphorylation-AMP-activated protein kinase (p-AMPK), carnitine palmitoyl transferase I (CPT-1), and Malonyl-CoA decarboxylase (MLYCD) were assessed in liver tissue utilizing Real-time PCR (q-PCR) and western blotting techniques.

Results After 6-week treatment, Yam Gruel could significantly reduce liver weight and index. The lipid profile concentrations (TG, TC, NEFA, and LDL-C) and liver function (ALT and AST) improved, as did the degree of hepatic steatosis in diabetic rats. Furthermore, Yam Gruel improved the protein levels of p-ACC/ACC, p-AMPK/AMPK, MLYCD, and CPT-1, consistent with gene expression trends.

Conclusions The results of the research demonstrated that Yam Gruel would be a potential therapy diet for improving lipid metabolism in T2DM through activating AMPK/ACC/CPT-1 pathway. In some respects, the combination therapy of Yam Gruel and Metformin showed greater benefits.

Keywords Yam Gruel, Diet therapy, Type 2 diabetes, Dyslipidemia, Liver, AMPK signaling pathway

Introduction

Diabetes is among the most rapidly expanding health problems of the 21st century,

distinguished via a substantial elevation within the prevalence of those affected by this condition tripling over the previous two decades. The International Diabetes Federation (IDF) has recently published the 9th edition of the Diabetes Atlas, 463 million individuals, or 9.3% of the population aged between 20 and 79, live with diabetes. Additionally, the IDF projects that by 2030 and 2045, the global population affected by diabetes is anticipated to reach 578 million and 700 million correspondingly [1]. An exponential increase in T2DM in developing countries, including China, is well recognized owing to the aging of the demographic and changing cultures [2]. Based on the Chinese guidelines for T2DM prevention and management (2020 Edition) [3], in China, the prevalence of this disease was estimated to be 11.2%, displaying a sustained upward trend. Meanwhile, those with diabetes have an elevated susceptibility to the onset of several significant health conditions. Constantly increased blood sugar levels can lead to significant disorders affecting the liver, heart, blood vessels, kidneys, and nerves. Notably, around 50% of the population affected by diabetes remains uninformed about their health status, placing them at considerable risk for developing severe and life-threatening problems [4,5]. Diabetes and its complications raise the demand for medical care, diminish the quality of life (QoL), and place an excessive burden on families. Consequently, T2DM imposes a substantial burden on healthcare resources, necessitating the development of effective therapies to combat T2DM epidemics.

Dyslipidemia is a distinct and reversible risk factor for T2DM, and individuals suffering from T2DM have impaired lipid metabolism [6,7]. Earlier researches have demonstrated that over half of T2DM patients exhibit dyslipidemia, a condition that

involves elevated concentrations of total triglyceride (TG), cholesterol (TC), and low-density lipoprotein cholesterol (LDL-C) or a reduction in high-density lipoprotein cholesterol (HDL-C) [8]. Dyslipidemia would result in the occurrence and development of cardiovascular disorder (CVD) in T2DM individuals [9]. Some studies have also demonstrated that a severe impairment in HDL-C function would increase CVD risk [10]. A multicenter nationwide survey, with 4807 Chinese adult patients from 20 endocrinology clinics in China, showed the considerable incidence of dyslipidemia (67.1%). However, the study also highlighted a notable lack of knowledge, treatment, and management measures in individuals suffering from type 2 diabetes (68.7%, 55.9%, and 39.5% respectively) [11]. Therefore, the influence of dyslipidemia on T2DM development and progression should not be overlooked, and effective methods should be quested for effective methods to relieve dyslipidemia in T2DM patients. In the present moment, statins are widely regarded as the primary option for lipid-lowering medications across various therapeutic contexts, but the use of statins is influenced by many factors, including safety and tolerability and patient adherence [12,13]. Chinese individuals have a higher susceptibility to the development of hepatotoxicity and muscle symptoms than others in Europe and America, even the long-term administration of statins leading to fluctuations in blood glucose [14]. Over the course of the previous ten years, a number of lipid-lowering medications that are not statins have come to the forefront， such as anti-PCSK9 monoclonal antibodies, but most of the new therapies are currently in the process of being subjected to experimental or clinical trials, as well as being not widely used due to their high cost and inconvenient

delivery method [15,16]. Therefore, the current guideline and expert consensus clearly stress that promoting healthy dietary and lifestyle adjustments is the primary therapeutic approach for dyslipidemia management in T2DM patients [14,17-19].

The most difficult aspect of the treatment regimen for many diabetic patients is deciding what to eat. Nutritional therapy is the cornerstone of T2DM care and performs a pivotal function in the comprehensive regulation of diabetes, even when medication has been initiated. The implementation of nutritional therapy in the management of diabetes has been shown to have positive effects such as weight reduction, the prevention of diabetic complications, and the attainment of favorable leads to glycemic control, blood pressure, and lipids, and glycosylated hemoglobin reduction as well as cost savings [20-24]. Based on the 2019 publication titled "Nutrition Therapy for Adults with Diabetes or Prediabetes: A Consensus Report," various eating regimens are appropriate for T2DM management, such as low-carbohydrate diets, Mediterranean diets and low-fat vegan diets [25-28].

However, it is important to note that there is no ideal and "one-size-fits-all" food for diabetic patients throughout the world, and planning the meals must be tailored depending on subjective and cultural preferences, resources, and health goals. Rice is an important carbohydrate crop providing a staple food to more than 65% of the population in China. Based on habitual dietary patterns, T2DM patients in China face the challenge of adapting to carbohydrate-restricted diets, especially for breakfast. Therefore, we attempt to replace traditional rice soup with Yam Gruel to lower carbohydrate intake.

Yam Gruel is a classic diet therapy prescription from Xichun Zhang, a famous doctor of Traditional Chinese medicine in the Ming Dynasty [29]. Yam Gruel contains exclusively Chinese Yam, which is not only a usual food in China with a long history but also a herb typically used for invigorating the spleen and stomach to regulate metabolism. In China, Yam Gruel is recommended as a healthy diet for T2DM patients. In accordance with an earlier investigation, the dietary intervention combined with Yam Gruel can reduce fasting blood glucose (FBG), 2 h postprandial blood glucose (2hPBG) levels, and improve blood lipid profile (namely TG, TC and LDL-C) within T2DM patients, consistent with the results of the animal experiments [30-31]. To some extent, the combination of Yam Gruel with metformin would bring more efficacy and advantages in the improvement of insulin sensitivity and decrease of FBG and TG [32,33]. It has been known that AMPK is a key regulatory molecule that improves hepatic insulin resistance and lipid metabolism. There has been much research showing that AMPK/ACC/CPT-1A pathway activation in liver stimulates the oxidation of free fatty acid, in order to decrease lipid accumulation [34,35]. Previous studies have proven that Yam Gruel could prevent insulin resistance and improve glucose metabolism via the activation of AMPK pathway within skeletal muscle and pancreas of type 2 diabetes mellitus rats [32,33] However, whether Yam Gruel can affect hepatic lipid metabolism through AMPK/ACC/CPT-1A pathway remains unknown.

Given the potential stimulation of the AMPK pathway within skeletal muscle and pancreas by Yam Gruel, along with its significant association with lipid metabolism, this study employed diabetic rats fed with Yam Gruel to evaluate its impact on serum

lipid levels, liver function, and hepatic steatosis. Additionally, the stimulation of the AMPK pathway within liver tissue underwent assessment using q-PCR and western blotting techniques. The current investigation findings reveal crucial discoveries into the mechanism by which Yam Gruel exerts its anti-lipid effects on diabetic rats.

Methodology

Yam Gruel Preparation

Yam Gruel was produced utilizing the methodologies outlined within the publication "Records of Traditional Chinese and Western Medicine in Combination" authored by Xichun Zhang [36].

During preparation, the skin was removed from 150 g of raw Chinese yam, then sliced, with each Chinese yam slice weighing 125 g. Each Chinese yam slice was placed together with 50 mL of water into a homogenizer to form a thick paste. The paste was placed in 250 mL volume of cold water and then heated on a low to medium heat setting for a duration of 30 s each time. The process of boiling has been conducted thrice at 30-s intervals. Throughout the boiling phase, the paste was carefully mixed. The final concentration of Yam Gruel is approximately 0.5 g/mL [31,37].

Animals and type 2 diabetes rat model

Forty SPF-grade male Wistar rats (8-week-old, 200 ± 20 g) have been procured from Shanghai SLAC Laboratory Animal Co., LTD (Shanghai, China). Following a period of 1 week adaptive breeding, 40 rats underwent random allocation into two separate groups: for six weeks, the control group (n=6) was provided with a basic food,

and T2DM model group (n=34) was provided with a fat-rich regimen (15% sucrose, 10% lard, 10% yolk powder, 4% cholesterol, 60.7% standard diet and 0.3% chocolate) for six weeks as well. After 6-week high-fat dieting followed by 12 h fasting, the rats within T2DM model group received a 25 mg/kg+m^2 of intraperitoneal streptozocin injection [STZ, freshly produced citrate buffer solution (pH 4.5)], with another intraperitoneal injection of STZ being performed after a 72-h interval. Meanwhile, the rats in the control group received a comparable volume (25 mg/kg+m^2) of citrate buffer. The fasting blood glucose levels were assessed on the third and seventh days following injection by extracting blood from the tail vein and utilizing a consistent, portable blood glucose meter. If the rat's post-fasting blood glucose levels within the model group were over 11.1 mmol/L, the modeling was deemed successful. Eventually, 24 successful T2DM and 6 control rats were chosen for intervention in subsequent studies. The Laboratory Animal Ethics Committee of Fujian University of Traditional Chinese Medicine approved the current study (FJTCM IACUC 2019056).

Animal regrouping and interventions

A total of thirty rats underwent random allocation to five distinct groups through the following manner: (1) control group (normal saline, 10 mL/kg/d, n=6), (2) model group (normal saline, 10 mL/kg/d, n=6), (3) SYZ group (Yam Gruel, 12 g/kg/day, n=6), (4) MET group (Metformin, 100 mg/kg/d, n=6), and (5) Combined group (Yam Gruel combined with Metformin, a dose of gavage was the same as above, n=6). The interventions in each group lasted for six weeks. Throughout the trial, each rat group's body weight was evaluated on a weekly basis to modify the gavage dose.

Tissue processing

Following 6 week treatment, the rats were subjected to anesthesia using a 2% solution of sodium pentobarbital (45 mg/kg) after 12 h overnight fasting. Blood from the abdominal aorta was drawn into BD Vacutainer® Venous Blood Collection Tubes (SST™ Serum Separation Tubes Becton, Dickinson and Company, Franklin Lakes, NJ, USA) and underwent centrifugation to extract the serum (3000 rpm, 10 min). The serum underwent freezing at a temperature of –80 °C for future processing. Complete liver tissue was removed, weighed, and then cut into smaller parts. Two pieces of liver tissues per sample have been preserved at a temperature of –80 °C for western blotting and q-PCR, while the two other pieces per sample underwent immediate freezing within liquid nitrogen for Oil Red O staining.

Serum biochemical analysis

TC, TG, HDL-C, LDL-C, non-esterified fatty acids (NEFA), alanine aminotransferase (ALT), and aspartic acid transaminase (AST) serum levels were determined following the instructions on the respective test kits. In short, all serum samples were thawed and subjected to centrifugation for a duration of 10 min again at 4 °C and 3000 rpm. A 15 μL of each serum sample was added to the Chemray800 Automatic Biochemistry Analyzer to measure the lipid profile and liver function levels.

Oil Red O staining in liver tissue

In order to evaluate hepatic steatosis, frozen liver slices 8-μm-thick were produced and subjected to staining utilizing Oil Red O. 0.5 g of Oil Red O (Sigma-Aldrich, St.

Louis, MO, USA) underwent dissolving using 100 mL volume of 98% isopropanol for creating saturated Oil red O staining. The Oil red O staining that was suitable for use has been produced through the process of dilution, whereby a saturated Oil red O solution was diluted by a ratio of 6:4 with distilled water and then filtered after standing for 10 min. Staining occurred, avoiding light at room temperature for a duration of 15 min. The tissue slices have been placed into 60% *ethanol* to continue color separation. Images were captured using a fluorescence microscope (Leica inverted fluorescence microscope DM IL LED, Leica, Wetzlar, Germany). Image Pro plus 6.0 software was utilized to determine the positive area and the number of lipid droplets.

Extraction of protein and western blot technique

The extraction of total protein from liver tissue in each group of rats has been conducted using RIPA Lysis Buffer (MA0151, meilunbio®, Dalian, China) at 4 °C, resolved on SDS-PAGE, and moved onto PVDF membranes. TBST, containing 5% skim milk, was used to block the membranes at a temperature of 37 °C for a duration of 1 h, subjected to incubation at a temperature of 4 °C overnight with specified antibodies, and then by appropriate HRP-conjugated secondary antibodies (1:1000 dilution) at normal temperature for a duration of 1 h. Primary antibodies were AMPK and ACC Antibody Sampler Kit (#9957 Cell Signaling Technology, Shanghai, China), DCMC rabbit pAb (YN4175, ImmunoWay Biotechnology Company, Texas, USA,), CPT1A rabbit pAb (YN3388, ImmunoWay Biotechnology Company, Texas, USA), and Anti-beta Actin Mouse mAb (GB12001, Wuhan Servicebio technology Co., LTD, Wuhan, China). Using enhanced chemiluminescence technique, immunoreactive

proteins were identified. Grayscale analysis was utilized to quantify protein expression with ImageJ software (version 6.0.0.260, Media Cybernetics Corporation, USA).

Real-time PCR analysis

According to the guidelines provided by the manufacturer, total RNA was extracted using the RNA Reagent (TIANGEN BIOTECH (BEIJING)Co., LTD., Beijing, China). Reverse transcription was performed in accordance with the handbook of NovoScript® 1st Strand cDNA Synthesis SuperMix (gDNA Purge) (Novoprotein Scientific Inc., Beijing, China). Prkaa2, Acaca, MLYCD, CPT1A, and Actb expression levels were quantified using an ABI Step one plus Real-Time PCR system (Thermo Fisher Scientific Inc. Massachusetts, USA) and NovoStart® SYBR qPCR Supermix Plus (Novoprotein Scientific Inc. China). The Prkaa2, Acaca, MLYCD, and CPT1A mRNA expression levels in each sample were normalized to the level of Actb. Table 1 shows the primers for these genes. Each experiment was conducted in duplicate. The guidelines for the cycling conditions were as follows: 10 min period at 95 °C, subsequent to 40 cycles of 15 s at 95 °C, and finally, a minute at 60 °C. The data obtained underwent calculation using the comparative Ct technique ($2^{-\Delta\Delta Ct}$ method), by which ΔΔCt = ΔCt sample – ΔCt.

Table 1 Primer sequences

Gene name	Forward primer	Reverse primer	Length(bp)
Prkaa2	TCCTTCATGGACGATATGGCC	ATCCAGTGGACAGCGTGCTTT	108
Acaca	GCGGCTCTGGAGGTATATGT	TTAGCGTGGGGATGTTCCCT	151

MLYCD	CAGGGAAAGGAGTATGGGAGG	TTCTCAGACTTCGCCCACTCA	134
CPT1A	CTTCCCCTTACTGGTTCC	ACTCTCCCGCTGTTGTCC	202
Actb	ACTCTGTGTGGATTGGTGGC	AGCTCAGTAACAGTCCGCCT	137

Statistical analysis

The data was examined utilizing SPSS 22.0 software (IBM Corp., Armonk, NY, USA) to represent the results as the mean ± standard error of the mean (SEM). One-way analysis of variance (ANOVA) was employed for assessing statistical significance between multiple groups, complied with the LSD-test (assuming equal variances) and Tamhane's T2-test (without the assumption of equal variances). A *P*-value under 0.05 is expressed as statistically significant.

Results

Influence of Yam Gruel on body weight and FBG in T2DM rats

Figure 1 shows that rats with diabetes had a significant reduction within the body and elevation within FBG after modeling ($P<0.01$), which indicated the successful establishment of T2DM models. In comparison to the model group, their FBG concentrations within the SYZ, MET and Combined groups declined from the fourth week and was significantly reduced by the sixth week after treatment ($P<0.01$). In the three intervention groups, the rats' final body weight increased, while within the model group, it reduced ($P<0.05$).

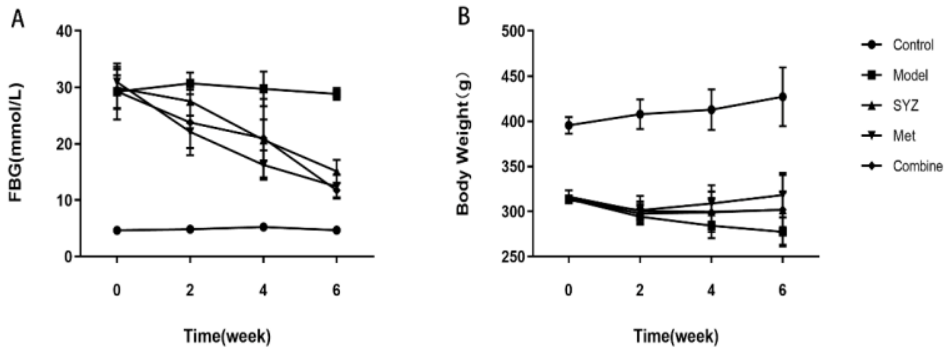

Fig.1 Impact of Yam Gruel on body weight and FBG within T2DM rats

Yam Gruel reduced liver's weight and index in T2DM rats

As revealed within Table 2, the weight of the liver and index (the liver-to-body weight ratio) of T2DM rats within the model group elevated significantly (*P*<0.01) in comparison to the control group. However, contrasted to the model group, the weight and index of the liver in rats with T2DM within the groups SYZ, MET, and Combined showed a significant reduction (*P*<0.01) after six weeks of gavage interventions. According to pairwise comparisons, no statistically significant variation found within the liver's weight and index between SYZ, MET, and Combined groups (*P*>0.05).

Table 2 Impact of Yam Gruel on liver weight and body in T2DM rats.

Group	Body weight (g)	Liver weight (g)	Liver index
Control	427.00±32.58	11.21±0.71	2.63±0.16
Model	277.33±16.02	15.51±1.28**	5.32±0.42**
SYZ	301.63±38.66	10.03±0.35$^{\#\#}$	3.37±0.42$^{*\#\#}$
MET	318.13±24.69	10.42±0.78$^{\#\#}$	3.28±0.16$^{**\#\#}$
Combine	301.86±19.30	10.04±0.60$^{\#\#}$	3.33±0.18$^{**\#\#}$

$^{*}P < 0.05$, $^{**}P < 0.01$, compared with the control group; $^{\#}P < 0.05$, $^{\#\#}P < 0.01$, compared with the model group.

Yam Gruel reduced lipid profile and improved liver function in T2DM rats

After six weeks of treatments, the lipid profile levels, including HDL-C, LDL-C, TC, TG and NFFA within serum were measured utilizing ELISA method. As displayed in Figure 2, the LDL-C, TC, TG and NEFA blood levels within the model group were significantly greater in comparison to the control group, whereas a significant decrease was found in the model group's serum HDL-C ($P<0.01$). Contrasted with the model group, serum concentrations of LDL-C, TG, TC and NEFA declined significantly, whereas serum HDL-C exhibited a notable increase in all three treatment groups ($P<0.01$). As shown in the results of biochemical analysis in Figure 3, the liver function was examined in serum, including AST and ALT. Their serum levels have been increased relative to the control group ($P<0.01$). Contrasted to the model group, serum ALT and AST concentrations were reduced in SYZ, MET, and Combined groups ($P<0.01$). Moreover, pairwise comparisons between the three therapy groups (SYZ, MET, and Combined) showed that the decrease within TC, LDL-C, NEFA, ALT, and AST serum levels in the combination group was substantially larger than in SYZ or MET groups ($P<0.05$, $P<0.01$, sequentially). The aforementioned outcomes proposed that Yam Gruel treatment has the potential to enhance serum lipid metabolism and liver function.

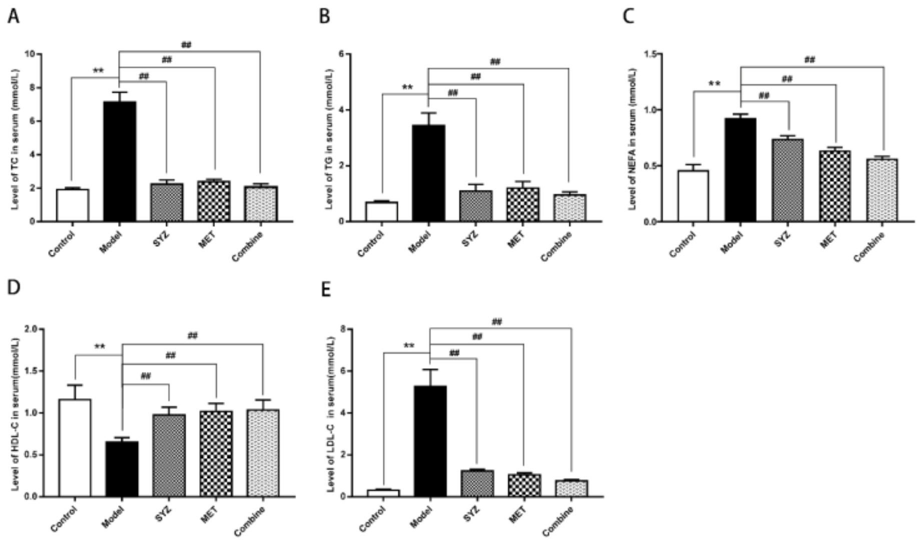

Fig.2 Yam Gruel decreased the lipid profile within diabetic rat's serum

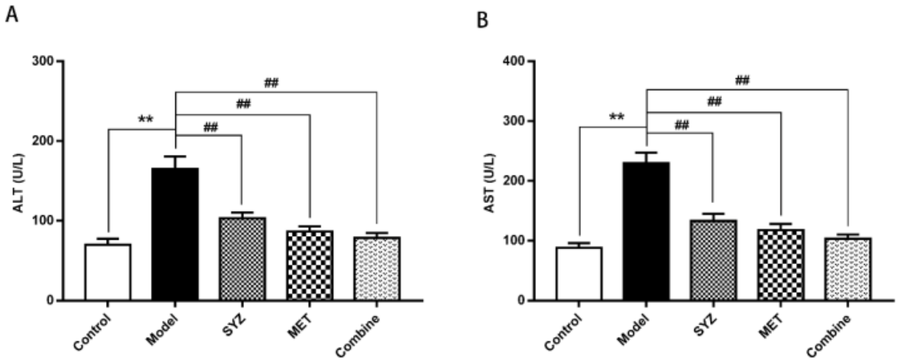

Fig.3 Yam Gruel improved liver function within diabetic rat's serum

Yam Gruel enhanced liver fat accumulation and pathological changes

First, each group's macro-morphologies of liver tissue were examined after six weeks of treatments. In a macroscopic view, the livers in the control group were a healthy reddish-brown color, with a soft and elastic appearance with a sharp edge, smooth surface, and normal size. Concurrently, the livers in the model group displayed

obvious enlargement and numerous spots of white fat accumulation. In comparison to the model group, the liver size and fat accumulation were substantially decreased in all three treatment groups.

Then, the histopathological alterations within the liver tissue of all groups were assessed. According to Figure 4, staining with Oil-red O uncovered that Yam Gruel, Metformin, and Combined treatments visibly attenuated the accumulation of hepatic lipids, as evidenced by fewer ballooning hepatocytes and intracellular lipid droplets. Contrasted to the control group, the area of positive Oil-red O staining within the model group remarkably elevated ($P<0.01$), whereas the positive area in SYZ, MET, and Combined groups all showed a significant reduction contrasted to the model group ($P<0.01$).

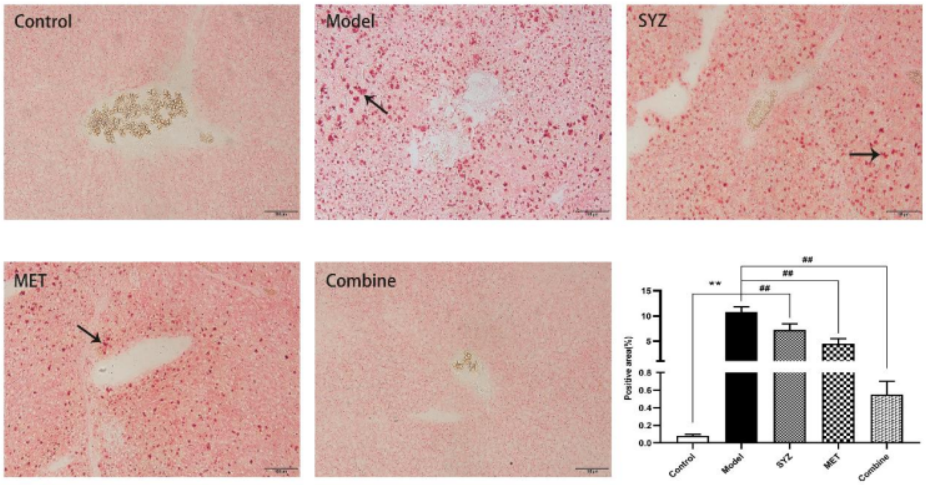

Fig.4 Impact of Yam Gruel on histopathological changes of liver tissues

Yam Gruel activated the AMPK/ACC/CPT-1 pathway in the tissue of the liver

For investigating the potential pathway through which Yam Gruel improves hepatic lipid metabolism in rats with T2DM, an investigation into the induction of the

AMPK/ACC/CPT-1 pathway was conducted. The relative mRNA levels of Prkaa2, MLYCD, and CPT1A exhibited significant reduction in the liver tissue of diabetic rats contrasted with those found within the control group; however, the mRNA expression level of Acaca exhibited significant elevation (P<0.01). However, treatment with Yam Gruel or Metformin for six weeks substantially restored the reduction across the mRNA expression of Prkaa2, MLYCD, and CPT1A, and the elevation across the mRNA expression level of Acaca (P <0.01), as represented in Figs. 5A–D.

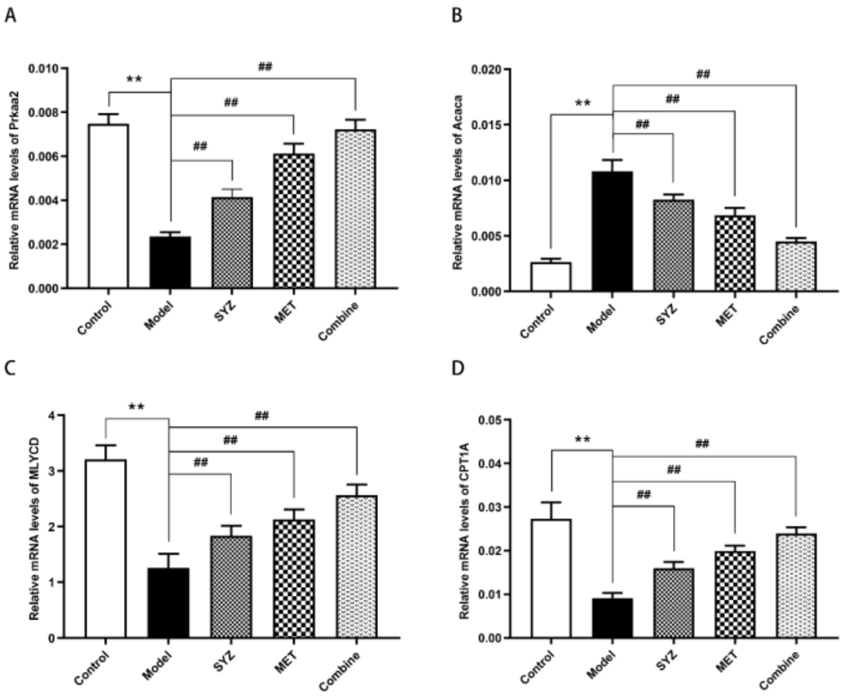

Fig.5 Impact of Yam Gruel on the mRNA expression of AMPK/ACC/CPT-1 pathway within liver tissue of T2DM rats.

As revealed in Figs. 6A–E, the protein levels of p-ACC/ACC, p-AMPK/AMPK, MLYCD, and CPT-1 exhibited significant decrease (P<0.01) in the livers of diabetes rats of the model group versus the control group. In contrast, protein expression levels

within rat's liver tissues across each of the three groups of treatment underwent significant elevation contrasted to those of rats within the control group (P<0.01 for all).

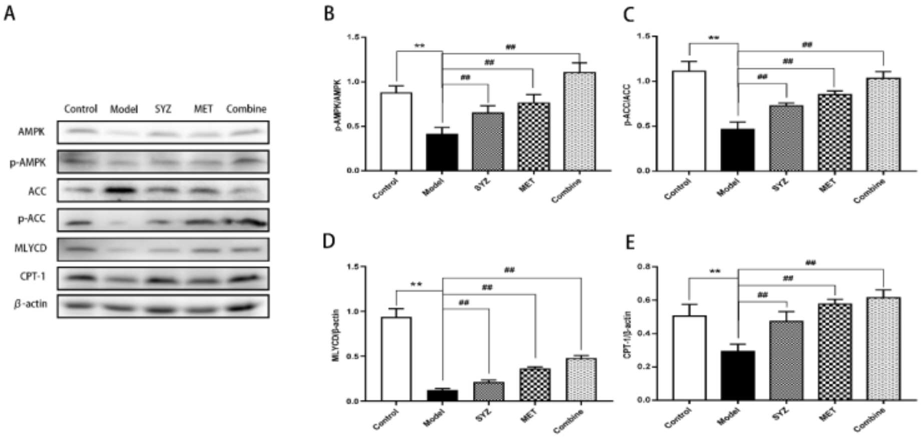

Fig.6 Impact of Yam Gruel on the protein expression of AMPK/ACC/CPT-1 pathway in liver tissue of T2DM rats.

Discussion

Drawing upon the principles of Traditional Chinese Medicine, dyslipidemia in T2DM patients is mainly owing to the disorder of spleen function, which could enhance the absorption and transportation of nutrient substances. In the recent years, theory of "Homogeny of Spleen and Pancreas" put forward by He Shaoqi (famous doctor of Traditional Chinese Medicine, TCM) [36], also support the perspective that spleen-qi deficiency would induce disturbance in the glucose and lipid metabolism. Many studies confirmed that spleen Qi and Yin deficiency was found to be the most common TCM syndrome among hyperlipidemia in T2DM patients [38]. So, strengthening spleen Qi and nourishing Yin is an important principle for the clinical treatment of abnormal glucose- lipid metabolism caused by T2DM. Previous meta-analysis showed that the

traditional Chinese dietary therapy based on supplementing Qi and Nourishing Yin Method could lower the level of FBG, 2hPBG, and HbA1c in T2DM patients [37]. Some experimental animal research has revealed that the “supplementing Qi and Nourishing Yin” prescription can significantly reduce liver index, blood lipid, and the extent of steatosis within rat’s liver tissues with induced diabetes using a combination of high-fat feed and STZ [30,39,40].

In the current study, Yam Gruel and metformin were selected as interventions for comparison in the treatment of T2DM rats. Yam Gruel is mainly made of Chinese Yam, which is a famous traditional food and medicine in China. It is widely distributed in China, but Jiaozuo in the Henan Province has become a genuine producing area due to its high quality. Consequently, Chinese Yam used in the study is produced from Jiaozuo. Chinese Yam is classified as “Qi-Invigorating Herbs”, and its main function is to strengthen spleen qi, and nourish spleen yin, which can effectively improve lipid and glucose metabolism and is one of the core medicines for the treatment of T2DM [41]. In modern pharmacology, the beneficial characteristics of Chinese Yam depends on its active ingredients. Over the past years, several *in vitro* and *in vivo* investigations have shown that these major bioactive ingredients are isolated from Chinese Yam, such as polysaccharides, diosgenin, polyphenols, allantoin, and starch, ameliorating glucose and lipid metabolism disorders in T2DM patients [42-44]. Metformin is the first choice for T2DM treatment in Chinese guideline of prevention and treatment T2DM [3]. It is known that metformin， a potent activator of AMPK, could inhibit hepatic glucose synthesis, improve lipid metabolism, and lower blood glucose [45].

Throughout the current investigation, compared with the control group, FBG and serum lipid profile concentrations (including TG, TC, LDL-C, and NEFA) all increased markedly across the diabetic rats. It means that T2DM rats show disorder of glucose and lipid metabolism obviously. It is found that feeding diabetic rats Yam Gruel not only result in a substantial reduction within FBG from the fourth to sixth week during the treatment, but also exhibited a notable decline in TC, TG, LDL-C, and NEFA levels, which are the important biochemical indicator representing lipid metabolism. Expert consensus points out that the decrease of LDL-C level is the fundamental objective of blood lipid regulation in patients diagnosed with type 2 diabetes [14.46]. After 6-week intervention, all three treatments lower the serum level of LDL-C by more than 50%. Because the role of liver in regulating lipid depends on its structure and function, the liver function and pathological alterations were evaluated. The outcomes show that the ALT and AST levels in diabetic rats were elevated relative to the control group. Concurrently, the livers within the model group displayed obvious enlargement and numerous spots of fat accumulation. These illustrated that diabetic rats had liver metabolic dysfunction and hepatocyte steatosis. Following up to 6 weeks of gavage, a significant reduction of ALT, AST and the area of positive Oil-red O staining solution have showed within the SYZ, MET, and Combine groups. In short, Yam Gruel or (and) metformin exhibits liver-protective effects, which would be the important structural basis of alleviating T2DM-related hyperlipidemia.

However, there has been no uniform conclusion on the mechanism of anti-diabetes and anti-lipid action of Yam Gruel thus far. Glucose-lipid homeostasis is most strongly

influenced by the liver, the center of energetic changes in the body, which contributes to the synthesis of fatty acid and cholesterol, oxidation of fatty acid. The ability of liver regulating lipid metabolism is not only dependent on normal tissue structure and function, but also controlled by multiple cellular signaling pathways. AMP-activated protein kinase (AMPK), contained one catalytic subunit (α1 or α2) and two regulatory subunits, has always been considered a potential target for maintaining energy homeostasis within the liver, muscle and adipose tissue [47]. AMPK is particularly closely linked to fatty acid oxidation and hepatic lipogenesis [48]. AMPK dephosphorylation causes sequential dephosphorylation of ACC, increasing malonyl-CoA and inhibiting CPT1A-dependent fatty acid oxidation. Malonyl-CoA decarboxylase (MLYCD), generating acetyl-CoA from malonyl-CoA, is activated by AMPK [49,50]. Xu et al. revealed that dioscin could decrease the levels of ACC gene in diet rich with fat and streptozotocin-stimulated T2DM rats, leading to a decrease in lipogenesis [51]. In a study on streptozotocin-induced diabetic rats, scientists demonstrated that 25 Callicarpa nudiflora extract might enhance oral glucose tolerance, lipid metabolism, insulin resistance, and cure diabetes-associated liver and pancreatic impairment through activating AMPK-ACC pathway [52]. In this study, the protein levels of p-ACC/ACC, p-AMPK/AMPK, MLYCD, and CPT-1 were reduced significantly within the livers of diabetes rats of the model group versus the control group. This result demonstrated that AMPK phosphorylation was attenuated and liver-specific AMPK pathway was suppressed in T2DM rats. Administration of Yam Gruel upregulated mRNA expression of Prkaa2, MLYCD, and CPT1A, as well as

downregulated that of Acaca. The protein levels of p-ACC/ACC, p-AMPK/AMPK, MLYCD, and CPT-1 were increased in the liver tissue, consistent with gene expression trends. It means that the impact of Yam Gruel on lipid metabolism recovery in diabetic rats may be mediated by activating AMPK signaling pathway.

Strengths and restrictions

The present investigation primarily examines the issue of diet treatment for patients with T2DM in China, with a comprehensive emphasize on the utilization of Yam Gruel as a substitute food to decrease carbohydrate consumption. The evaluation of the impact of Yam Gruel on liver function and liver pathological alterations is conducted for the first time within this research. Furthermore, the induction of the hepatic AMPK/ACC/CPT-1 pathway suggests that Yam Gruel may serve as a potential therapeutic diet for enhancing lipid metabolism across patients with T2DM.

However, it is critical to acknowledge that this research is not without its limitations. While the findings of this study do support the notion that Yam Gruel can enhance lipid metabolism in diabetic rats through inducing the AMPK pathway in the liver, the specific bioactive compounds and metabolic constituents responsible for its lipid-lowering and hepatoprotective effects remain unclear. Therefore, further comprehensive and extensive investigations are warranted to shed light on these aspects in future research endeavors.

Conclusion

This study reveals that Yam Gruel treatment markedly reduced the liver index and

weight, hepatic steatosis, and fat accumulation in high-fat along with STZ- stimulated diabetic rats. The activation of AMPK/ACC/CPT-1 pathway appears to be the mediator of these effects. Furthermore, the combination therapy of Yam Gruel and Metformin would achieve greater benefits for relieving dyslipidemia in T2DM. The inclusion of Yam Gruel in the dietary regimen for diabetes patients in China may effectively address the issue of excessive carbohydrate consumption, while also mitigating lipid metabolism disorders and liver damage, thereby potentially serving as a viable dietary intervention for dyslipidemia associated with type 2 diabetes mellitus.

Figure Legends

Fig.1 Impact of Yam Gruel on body weight and FBG within T2DM rats. **(A)** the changes of FBG of rats within all groups throughout the 6-week intervention; **(B)** rat's body weight changes within all groups throughout the 6-week intervention.

Fig.2 Yam Gruel decreased the lipid profile levels in the diabetic rats. **(A)** the level of total cholesterol (TC) in the rats across all groups; **(B)** the level of triglyceride (TG) in the rats across all groups; **(C)** the level of nonesterified fatty acids (NFFA) in the serum of rats across all groups; **(D)** HDL-C in the serum of rats across all groups; **(E)** LDL-C level within the serum of rats in each group; $^{**}P$<0.01, contrasted to the control group; $^{\#\#}P$<0.01, contrasted to model group. The data are expressed as mean ± SD (n = 6).

Fig.3 Yam Gruel improved liver function in the serum of diabetic rats. **(A)** Alanine aminotransferase (ALT) within rat's serum across all groups; **(B)** aspartic acid transaminase (AST) in the serum of rats in each group. $^{**}P$<0.01, contrasted to control group; $^{\#\#}P$<0.01, contrasted to model group. The data was reported as mean ± SD (n = 6).

Fig.4 Impact of Yam Gruel on histopathological alterations of liver tissues from diabetic rats after 6-week intervention. Microscopic images with 200 × total magnification. Scale bar: 100 µm. The data was presented as mean ± SD (n = 6).

Fig.5 Impact of Yam Gruel on the mRNA expression of AMPK/ACC/CPT-1 pathway in liver tissue of T2DM rats. (**A**) The relative mRNA level of Prkaa2; (**B**) the relative mRNA level of Acaca; (**C**) the relative mRNA level of MLYCD; (**D**) the relative mRNA level of CPT1A. $^{**}P<0.01$, contrasted to control group; $^{\#\#}P<0.01$, contrasted to model group. The data was reported as mean ± SD (n = 6).

Fig.6 Impact of Yam Gruel on AMPK/ACC/CPT-1 protein expression pathway within T2DM rats' liver tissues. (**A**) The findings of Western blot; (**B**) the protein expression level of p-AMPK/AMPK; (**C**) the protein level of p-ACC/ACC; (**D**) the protein level of MCoA/β-actin; (**E**) the protein level of CPT-1/β-actin. $^{**}P<0.01$, contrasted to control group; $^{\#\#}P<0.01$, contrasted to model group. The data was presented as mean ± SD (n = 6).

Declarations

Authors' contributions

Yanling Dai and Weinan Liu designed and guided the experiments; Yanling Dai wrote the original draft; Yanling Dai, Weinan Liu, Chen Qiu and Mianli Li conducted the investigations; Diandian Zhang examined the data. Weinan Liu revised the manuscript.

Funding

This investigation granted financial assistance from the National Natural Science Foundation of China (Grant Number: 81904269) and the Natural Science Foundation of Fujian Province (Grant Number: 2019J01358)

Data and materials availability

The data pertaining to this investigation is accessible upon demand.

Declarations of ethical approval and participation consent

All animal experiments granted approval from the Laboratory Animal Ethics Committee of Fujian University of Traditional Chinese Medicine (FJTCM IACUC 2019056).

Consent for publication Not applicable.

Competing interests The authors do not have any conflicting interests to disclose.

Author details :

[1] School of Nursing, Fujian University of Traditional Chinese Medicine, Fuzhou 350122, China;

[2] Department of Endocrine, People's Hospital Affiliated to Fujian University of Traditional Chinese Medicine, Fuzhou 350004, China;

[3] Department of Orthopedics, People's Hospital Affiliated to Fujian University of Traditional Chinese Medicine, Fuzhou 350004, China.

References

1. IDF. IDF Diabetes Atlas, 9th edn. Brussels, Belgium: International Diabetes Federation 2019;2.
2. Magliano DJ, Islam RM, Barr ELM, et al. Trends in incidence of total or type 2 diabetes: systematic review. BMJ. 2019;11(366): l5003.
3. Chinese Diabetes Society. Guideline for the prevention and treatment of type 2 diabetes mellitus in China (2020 edition). Chin J Diabetes Mellitus 2021;13(4):315-409.
4. Einarson TR, Acs A, Ludwig C, Panton UH. Prevalence of cardiovascular

disease in type 2 diabetes: a systematic literature review of scientific evidence from across the world in 2007-2017. Cardiovasc Diabetol 2018;17(1):83

5. ADA. Economic Costs of Diabetes in the US in 2017. Diabetes care 2018; 41:917.

6. Imai Y, Cousins RS, Liu S, Phelps BM, Promes JA. Connecting pancreatic islet lipid metabolism with insulin secretion and the development of type 2 diabetes. Ann N Y Acad Sci 2020;1461(1):53-72.

7. Chatterjee S, Khunti K, Davies MJ. Type 2 diabetes. Lancet 2017;389(10085): 2239-2251.

8. Jisieike-Onuigbo NN, Unuigbe EI, Oguejiofor CO. Dyslipidemias in type 2 diabetes mellitus patients in Nnewi South-East Nigeria. Ann Afr Med 2011;10(4):285-9.

9. Iqbal J, Al Qarni A, Hawwari A, Alghanem AF, Ahmed G. Metabolic Syndrome, Dyslipidemia and Regulation of Lipoprotein Metabolism. Curr Diabetes Rev 2018;14(5):427-433.

10. Lüscher TF, Landmesser U, von Eckardstein A, Fogelman AM. High-density lipoprotein: vascular protective effects, dysfunction, and potential as therapeutic target. Circ Res 2014;114(1):171-82.

11. Yan L, Xu MT, Yuan L, et al. Prevalence of dyslipidemia and its control in type 2 diabetes: A multicenter study in endocrinology clinics of China. J Clin Lipidol 2016;10(1):150-60.

12. Du Z, Qin Y. Dyslipidemia and Cardiovascular Disease: Current Knowledge,

Existing Challenges, and New Opportunities for Management Strategies. J Clin Med 2023;12(1):363.

13. Collins R, Reith C, Emberson J, et al. Interpretation of the evidence for the efficacy and safety of statin therapy. Lancet 2016;388(10059):2532-2561.

14. LI Q. Expert Consensus on Prevention and Treatment of Type 2 diabetes complicated with dyslipidemia in China (Revised Edition 2017). Chin J Endocrinol Metab 2017;33(11):925-936.

15. Tokgözoğlu L, Libby P. The dawn of a new era of targeted lipid-lowering therapies. Eur Heart J 2022;43(34):3198-3208.

16. Nishikido T, Ray KK. Non-antibody Approaches to Proprotein Convertase Subtilisin Kexin 9 Inhibition: siRNA, Antisense Oligonucleotides, Adnectins, Vaccination, and New Attempts at Small-Molecule Inhibitors Based on New Discoveries. Front Cardiovasc Med 2018; 5:199.

17. Grundy SM, Stone NJ, Bailey AL, et al. 2018 AHA/ACC/AACVPR/AAPA/ ABC/ACPM/ADA/AGS/APhA/ASPC/NLA/PCNA Guideline on the Management of Blood Cholesterol: A Report of the American College of Cardiology/American Heart Association Task Force on Clinical Practice Guidelines. J Am Coll Cardiol 2019;73(24): e285-e350.

18. Virani SS, Morris PB, Agarwala A, et al. 2021 ACC Expert Consensus Decision Pathway on the Management of ASCVD Risk Reduction in Patients With Persistent Hypertriglyceridemia: A Report of the American College of Cardiology Solution Set Oversight Committee. J Am Coll Cardiol

2021;78(9):960-993.

19. Agarwala A, Petersen KS, Jafari F, Kris-Etherton PM. Dietary management of dyslipidemia and the impact of dietary patterns on lipid disorders. Prog Cardiovasc Dis 2022; 75:49-58.

20. Evert AB, Dennison M, Gardner CD, et al. Nutrition Therapy for Adults with Diabetes or Prediabetes: A Consensus Report. Diabetes Care 2019;42(5):731-754.

21. Franz MJ, MacLeod J, Evert A, et al. Academy of Nutrition and Dietetics Nutrition Practice Guideline for Type 1 and Type 2 Diabetes in Adults: Systematic Review of Evidence for Medical Nutrition Therapy Effectiveness and Recommendations for Integration into the Nutrition Care Process. J Acad Nutr Diet 2017;117(10):1659-1679.

22. Parker AR, Byham-Gray L, Denmark R, Winkle PJ. The effect of medical nutrition therapy by a registered dietitian nutritionist in patients with prediabetes participating in a randomized controlled clinical research trial. J Acad Nutr Diet 2014;114(11):1739-48.

23. **MacLeod J, Franz MJ, Handu D, et al. Academy of Nutrition and Dietetics Nutrition Practice Guideline for Type 1 and Type 2 Diabetes in Adults: Nutrition Intervention Evidence Reviews and Recommendations. J Acad Nutr Diet**

2017;117(10):1637-1658.

24. Schwingshackl L, Schwedhelm C, Hoffmann G, et al. Food groups and risk of all-cause mortality: a systematic review and meta-analysis of prospective studies. Am J Clin Nutr 2017;105(6):1462-1473.

25. Finicelli M, Squillaro T, Di Cristo F, et al. Metabolic syndrome, Mediterranean diet, and polyphenols: Evidence and perspectives. J Cell Physiol 2019;234(5):5807-5826.

26. Estruch R, Ros E, Salas-Salvadó J, et al. Primary Prevention of Cardiovascular Disease with a Mediterranean Diet Supplemented with Extra-Virgin Olive Oil or Nuts. N Engl J Med 2018;378(25): e34.

27. The Diabetes Prevention Program (DPP): description of lifestyle intervention. Diabetes Care 2002;25(12):2165-71.

28. Bloomfield HE, Koeller E, Greer N, MacDonald R, Kane R, Wilt TJ. Effects on Health Outcomes of a Mediterranean Diet With No Restriction on Fat Intake: A Systematic Review and Meta-analysis. Ann Intern Med 2016;165(7):491-500.

29. Xichun Z. Traditional Chinese and Western Medicine in Combination: Chemical Industry Press, 2018:73-74.

30. Pang SQ, Li WT, Lin J, et al. Influence of Dioscorea opposita porridge on blood lipid in patients with type 2 diabetes mellitus. Nursing Research of China 2017;31(16):1941-1946.

31. Lin X, Luo Z, Pang S, et al. The effects of yam gruel on lowering fasted blood glucose in T2DM rats. Open Life Sci 2020;15(1):763-773.

32. DAI YL, CHEN XH, DING HL, LI ML, PANG SQ, WN L. EFFECTS OF SHUYU GRUEL (YAM GRUEL) ON THE GLUCOSE METABOLISM AND ISLET FUNCTION OF TYPE 2 DIABETIC MODEL RATS. JOURNAL OF

TRADITIONAL CHINESE MEDICINE 2021;62(18):1628-1634.

33. Lin XJ, Z J, Pang SQ, et al. Effects of Yam Gruel on AMPK signaling pathway and LC3-Ⅱ/p62 of autophagy in pancreas of type 2 diabetes mellitus rats, China Journal of Traditional Chinese Medicine and Pharmacy 2022;37(03):1326-1330.

34. Fang K, Wu F, Chen G, et al. Diosgenin ameliorates palmitic acid-induced lipid accumulation via AMPK/ACC/CPT-1A and SREBP-1c/FAS signaling pathways in LO2 cells. BMC Complement Altern Med 2019;19(1):255.

35. Tian X, Ru Q, Xiong Q, Wen R, Chen Y. Catalpol Attenuates Hepatic Steatosis by Regulating Lipid Metabolism via AMP-Activated Protein Kinase Activation. Biomed Res Int 2020; 2020:6708061.

36. Chao J, Liu Y, Zhong W, Fang C, Xie C, H G. Treating Diabetes Mellitus from the Viewpoint of Spleen: Based on the Theory of “Homogeny of Spleen and Pancreas”. Journal of Traditional Chinese Medicine 2017;58(17):1458-1461.

37. Dai YL, Liu WN, Pang SQ, Yang L, Li ML. Effects of traditional Chinese dietary therapy on blood glucose and quality of life on patients with type 2 diabetes mellitus: a systematic review. Journal of Guangxi University of Chinese Medicine 2019;22(01):140-146.

38. Chen XW, Tang SJ, YU M. Progress of TCM clinical research on type 2 diabetes mellitus complicated with abnormal lipid metabolism. Xinjiang Journal of Traditional Chinese Medicine 2021;39(03):119-122.

39. Ren Z, Dai PF, Liu Y, Zhang J, Mu X, Liu GL. Effect of Yiqi Yangyin Prescription on Lipid Metabolism in Rats with Type 2 Diabetes. Chinese Journal of Experimental Traditional Medical Formulae 2021;27(07):57-65.

40. Luo ZT, Pang SQ, Zhou J, Chen F, Hong XP, Chen JX, et al. Effects of Yam Gruel on Hepatic Gluconeogenesis of Type 2 Diabetic Rats. Journal of Hunan University of Chinese Medicine 2021;41(01):46-52.

41. Liu X, Qu C, Wang ST, Shen XH, Yang YF, Shi Y. Medication Rule of Chinese Medicine Treatment for Type 2 Diabetes Based on Data Mining. CHINESE ACHIVES OF TRADITIONAL CHINESE MEDICINE 2020;38(05):74-78.

42. Huang R, Xie J, Yu Y, Shen M. Recent progress in the research of yam mucilage polysaccharides: Isolation, structure and bioactivities. Int J Biol Macromol 2020; 155:1262-1269.

43. Go HK, Rahman MM, Kim GB, et al. Antidiabetic Effects of Yam (Dioscorea batatas) and Its Active Constituent, Allantoin, in a Rat Model of Streptozotocin-Induced Diabetes. Nutrients 2015;7(10):8532-44.

44. Cheng Z, Hu M, Tao J, et al. The protective effects of Chinese yam polysaccharide against obesity-induced insulin resistance. Journal of Functional Foods 2019; 55:238-247.

45. Wang J, Chakrabarty S, Bui Q, Ruf W, Samad F. Hematopoietic tissue factor-protease-activated receptor 2 signaling promotes hepatic inflammation and contributes to pathways of gluconeogenesis and steatosis in obese mice. Am J Pathol 2015;185(2):524-35.

46. ADA. Cardiovascular Disease and Risk Management: Standards of Medical Care in Diabetes-2021. Diabetes Care 2021;44(Suppl 1): S125-s150.

47. Zhang BB, Zhou G, Li C. AMPK: an emerging drug target for diabetes and the metabolic syndrome. Cell Metab 2009;9(5):407-16.

48. **Hardie DG. Sensing of energy and nutrients by AMP-activated protein kinase. Am J Clin Nutr 2011;93(4):891s-6.**

49. Foster DW. Malonyl-CoA: the regulator of fatty acid synthesis and oxidation. J Clin Invest 2012;122(6):1958-9.

50. Derdak Z, Villegas KA, Harb R, Wu AM, Sousa A, Wands JR. Inhibition of p53 attenuates steatosis and liver injury in a mouse model of non-alcoholic fatty liver disease. J Hepatol 2013;58(4):785-91.

51. XU LN, YIN LH, JIN Y, ET AL. EFFECT AND POSSIBLE MECHANISMS OF DIOSCIN ON AMELIORATING

METABOLIC GLYCOLIPID METABOLIC DISORDER IN TYPE-2-DIABETES. PHYTOMEDICINE 2020; 67:153139.

52. Ma WY, Ma LP, Yi B, Zhang M, Feng SX, Tian LP. Antidiabetic activity of Callicarpa nudiflora extract in type 2 diabetic rats via activation of the AMPK-ACC pathway. Asian Pacific Journal of Tropical Biomedicine 2019;9(11):456.

No.1236 5334words 20230829 Reduction.docx

ORIGINALITY REPORT

14%
SIMILARITY INDEX

PRIMARY SOURCES

1	www.frontiersin.org<br>Internet	89 words — 1%
2	www.mdpi.com<br>Internet	73 words — 1%
3	imrpress.com<br>Internet	48 words — 1%
4	www.spandidos-publications.com<br>Internet	29 words — < 1%
5	worldwidescience.org<br>Internet	25 words — < 1%
6	www.dovepress.com<br>Internet	23 words — < 1%
7	docksci.com<br>Internet	22 words — < 1%
8	www.degruyter.com<br>Internet	22 words — < 1%
9	hyper.ahajournals.org<br>Internet	21 words — < 1%
10	www.nature.com<br>Internet	

21 words — < 1%

11 Guanqun Chen, Pengbo Xu, Jian Pan, Yang Li, Junhui Zhou, Huiyun Kuang, Hongli Lian. "Inhibition of FvMYB10 transcriptional activity promotes color loss in strawberry fruit", Plant Science, 2020
Crossref
20 words — < 1%

12 www.jpsr.pharmainfo.in
Internet
19 words — < 1%

13 www.researchsquare.com
Internet
19 words — < 1%

14 Xian-pei Heng. "Strategies and methods for the treatment of diabetic neuropathy using integrative Chinese and Western medicine", Chinese Journal of Integrative Medicine, 12/2008
Crossref
17 words — < 1%

15 www.termedia.pl
Internet
16 words — < 1%

16 transmedcomms.biomedcentral.com
Internet
14 words — < 1%

17 www.jstage.jst.go.jp
Internet
14 words — < 1%

18 "The 21st Conference of the Asian Pacific Association for the Study of the Liver", Hepatology International, 2011
Crossref
13 words — < 1%

19 fbscience.com
Internet
13 words — < 1%

20 translational-medicine.biomedcentral.com
Internet
13 words — < 1%

21 Lei Zhao, Fei Pan, Na Zhou, Huimin Zhang, Yong Wang, Shuai Hao, Chengtao Wang. "Quantitative proteomics and bioinformatics analyses reveal the protective effects of cyanidin-3-O-glucoside and its metabolite protocatechuic acid against 2-amino-3-methylimidazo[4,5-f]quinoline (IQ)-induced cytotoxicity in HepG2 cells via apoptosis-related pathways", Food and Chemical Toxicology, 2021
Crossref
12 words — < 1%

22 Wan Hua, Shiyan Zhang, Qiuxia Lu, Yiran Sun, Shancai Tan, Fang Chen, Lin Tang. "Protective effects of n-Butanol extract and iridoid glycosides of Veronica ciliata Fisch. Against ANIT-induced cholestatic liver injury in mice", Journal of Ethnopharmacology, 2021
Crossref
12 words — < 1%

23 Wenling Tu, Yinjie Hong, Miaoan Huang, Meimei Chen, Huijuan Gan. "Effect of kaempferol on hedgehog signaling pathway in rats with - - chronic atrophic gastritis – Based on network pharmacological screening and experimental verification", Biomedicine & Pharmacotherapy, 2022
Crossref
12 words — < 1%

24 care.diabetesjournals.org
Internet
12 words — < 1%

25 oaji.net
Internet
12 words — < 1%

26 topsecretapiaccess.dovepress.com
Internet
12 words — < 1%

27 Gabrielle Nicole Gaultier, William McCready, Marina Ulanova. "The effect of pneumococcal immunization on total and antigen-specific B cells in patients with severe chronic kidney disease", BMC Immunology, 2019
Crossref — 11 words — < 1%

---

28 Jian Huang, Xuejun Chai, Yongji Wu, Yan Hou et al. "β‐Hydroxybutyric acid attenuates heat stress‐induced neuroinflammation via inhibiting TLR4/p38 MAPK and NF‐κB pathways in the hippocampus", The FASEB Journal, 2022
Crossref — 11 words — < 1%

---

29 coek.info
Internet — 11 words — < 1%

---

30 journals.plos.org
Internet — 11 words — < 1%

---

31 www.jove.com
Internet — 11 words — < 1%

---

32 Omnia Hamdy Abdel-Karim, Atef Mohamed Abo-Shady, Gehan Ahmed Ismail, Saly Farouk Gheda. " Potential effect of acetone extract on the biochemical and histological parameters of alloxan-induced diabetic rats ", International Journal of Environmental Health Research, 2021
Crossref — 10 words — < 1%

---

33 respiratory-research.biomedcentral.com
Internet — 10 words — < 1%

---

34 ressources.ciheam.org
Internet — 10 words — < 1%

---

35 www.cellsignaling.com.cn
Internet — 10 words — < 1%

36 www.science.gov
Internet
10 words — < 1%

37 Qi Li, Qiuping Huang, Ting Huyan, Yilin Wang, Qingsheng Huang, Junling Shi. "Bifacial effects of engineering tumour cell-derived exosomes on human natural killer cells", Experimental Cell Research, 2017
Crossref
9 words — < 1%

38 Ruohong Chen, Xingfei Lai, Limin Xiang, Qiuhua Li et al. "Aged green tea reduces high-fat diet-induced fat accumulation and inflammation via activating the AMP-activated protein kinase signaling pathway", Food & Nutrition Research, 2022
Crossref
9 words — < 1%

39 Zhang, Yi, Qian Qian, Dandan Ge, Yuhong Li, Xinrui Wang, Qiu Chen, Xiumei Gao, and Tao Wang. "Identification of Benzophenone <i>C</i>-Glucosides from Mango Tree Leaves and Their Inhibitory Effect on Triglyceride Accumulation in 3T3-L1 Adipocytes", Journal of Agricultural and Food Chemistry, 2011.
Crossref
9 words — < 1%

40 mdpi-res.com
Internet
9 words — < 1%

41 www.sciengine.com
Internet
9 words — < 1%

42 Longlong Li, Xu Chu, Yao Yao, Ji Cao, Qian Li, Haitian Ma. "(−)-Hydroxycitric Acid Alleviates Oleic Acid-Induced Steatosis, Oxidative Stress, and Inflammation in Primary Chicken Hepatocytes by Regulating AMP-Activated Protein Kinase-Mediated Reactive Oxygen Species Levels", Journal of Agricultural and Food Chemistry, 2020
8 words — < 1%

Crossref

43 Song, Xianbing, Hongsheng Liu, Xiaotian Wang, Zhenhua Li, and Congwu Huang. "Atorvastatin combined with poly-unsaturated fatty acid confers better improvement of dyslipidemia and endothelium function", Lipids in Health and Disease, 2014. — 8 words — < 1%
Crossref

44 Wang, Zhong-Chao, Du E, De-Ligen Batu, Ya-Latu Saixi, Bin Zhang, and Li-Qun Ren. "2D-DIGE Proteomic Analysis of Changes in Estrogen/Progesterone-Induced Rat Breast Hyperplasia upon Treatment with the Mongolian Remedy RuXian-I", Molecules, 2011. — 8 words — < 1%
Crossref

45 Yuxiang Pan, Cong Wang, Zhongqin Chen, Weiwei Li, Guoqi Yuan, Haixia Chen. "Physicochemical properties and antidiabetic effects of a polysaccharide from corn silk in high-fat diet and streptozotocin-induced diabetic mice", Carbohydrate Polymers, 2017 — 8 words — < 1%
Crossref

46 dokumen.pub — 8 words — < 1%
Internet

47 downloads.hindawi.com — 8 words — < 1%
Internet

48 findresearcher.sdu.dk:8443 — 8 words — < 1%
Internet

49 www.ncbi.nlm.nih.gov — 8 words — < 1%
Internet

50 www.pjps.pk — 8 words — < 1%
Internet

51 www.spkx.net.cn
Internet
8 words — < 1%

52 Abba Aji Manu, Bello Muhammad Musa, Martha Orendu Oche Attah, Helga Ishaya Bedan. "Ethanolic Extract of Syzygium cumini Causes Toxic Effects on Ethanol-induced Liver and Kidney Damage in Albino Wistar Rats: A Biochemical and Histological Study", Iranian Journal of Toxicology, 2022
Crossref
7 words — < 1%

53 Iwata, T.. "Effects of overexpression of basic helix-loop-helix transcription factor Dec1 on osteogenic and adipogenic differentiation of mesenchymal stem cells", European Journal of Cell Biology, 20060503
Crossref
7 words — < 1%

54 Jing Qu, Shuai Dang, Yuan-Yuan Sun, Tao zhang, Hai Jiang, Hong-Zhao Lu. "METTL21C mediates the occurrence of autophagy and formation of slow-twitch muscle fibers after exercise", Research Square Platform LLC, 2023
Crossref Posted Content
7 words — < 1%

55 Lee, Lee, Jung, Kwon, Kim, Hwang, Kim, Lee, Kang. "The Inhibitory Effect of Cordycepin on the Proliferation of MCF-7 Breast Cancer Cells, and its Mechanism: An Investigation Using Network Pharmacology-Based Analysis", Biomolecules, 2019
Crossref
7 words — < 1%

56 Petersen, S.L.. "Cytokine Gene Expression in Peripheral Blood Mononuclear Cells and Alloreactivity in Hematopoietic Cell Transplantation with Nonmyeloablative Conditioning", Biology of Blood and Marrow Transplantation, 200601
Crossref
7 words — < 1%

57 "Structure and Health Effects of Natural Products on Diabetes Mellitus", Springer Science and Business Media LLC, 2021 — 6 words — < 1%
Crossref

58 Kailin Yang, Tingting Bao, Jinsong Zeng, Shanshan Wang, Xiao Yuan, Wang Xiang, Hao Xu, Liuting Zeng, Jinwen Ge. "Research progress on pyroptosis-mediated immune-inflammatory response in ischemic stroke and the role of natural plant components as regulator of pyroptosis: A review", Biomedicine & Pharmacotherapy, 2023 — 6 words — < 1%
Crossref

59 Lijuan Mei, Qingyue Chen, Li Ge, Guohua Zheng, Jinxiu Chen. "Systematic Review of Chinese Traditional Exercise Baduanjin Modulating the Blood Lipid Metabolism", Evidence-Based Complementary and Alternative Medicine, 2012 — 6 words — < 1%
Crossref

60 Qin Kong, Haojun Zhang, Tingting Zhao, Weiku Zhang, Meihua Yan, Xi Dong, Ping Li. "Tangshen formula attenuates hepatic steatosis by inhibiting hepatic lipogenesis and augmenting fatty acid oxidation in db/db mice", International Journal of Molecular Medicine, 2016 — 6 words — < 1%
Crossref

61 Rodriguez, A.. "Impaired adiponectin-AMPK signalling in insulin-sensitive tissues of hypertensive rats", Life Sciences, 20081010 — 6 words — < 1%
Crossref

EXCLUDE QUOTES	OFF	EXCLUDE SOURCES	OFF
EXCLUDE BIBLIOGRAPHY	ON	EXCLUDE MATCHES	OFF
