# Supplementary material for: Yam Gruel alone and in combination with metformin regulates hepatic lipid metabolism disorders in a diabetic rat model by activating the AMPK/ACC/CPT-1 pathway
Source: Lipids Health Dis. 2024 Jan 25;23:28. doi: 10.1186/s12944-024-02014-2 (PMC10809441; doi:10.1186/s12944-024-02014-2)
Supplement: Supplementary file 3 — Supplementary Material 3 [file 12944_2024_2014_MOESM3_ESM.pdf]

Editing Certificate

This document certifies that the manuscript

Yam Gruel alone and in combination with metformin regulates hepatic lipid metabolism disorders in a diabetic rat model by activating the AMPK/ACC/CPT-1 pathway

prepared by the authors

Yanling Dai, Chen Qiu, Dian dian Zhang, Mian liLi, WeinanLiu

was edited for proper English language, grammar, punctuation, spelling, and overall style by one or more of the highly qualified native English speaking editors at AJE.

This certificate was issued on **December 19, 2023** and may be verified on the AJE website using the verification code **F596-162E-7CAD-42F9-9D58** .

Neither the research content nor the authors' intentions were altered in any way during the editing process. Documents receiving this certification should be English-ready for publication; however, the author has the ability to accept or reject our suggestions and changes. To verify the final AJE edited version, please visit our verification page at aje.com/certificate. If you have any questions or concerns about this edited document, please contact AJE at support@aje.com.

AJE provides a range of editing, translation, and manuscript services for researchers and publishers around the world.
For more information about our company, services, and partner discounts, please visit aje.com.
